# Synthesis and Modification of Morphine and Codeine, Leading to Diverse Libraries with Improved Pain Relief Properties

**DOI:** 10.3390/pharmaceutics15061779

**Published:** 2023-06-20

**Authors:** Mona Kamelan Zargar Zarin, Wim Dehaen, Peyman Salehi, Amir Ata Bahmani Asl

**Affiliations:** 1 Department of Phytochemistry, Medicinal Plants and Drugs Research Institute, Shahid Beheshti University, Evin, Tehran 1983963113, Iran; kamelan.zz_m@yahoo.com (M.K.Z.Z.); amiratachemistry.74@gmail.com (A.A.B.A.); 2 Sustainable Chemistry for Metals and Molecules, Department of Chemistry, KU Leuven, Celestijnenlaan 200F, B-3001 Leuven, Belgium

**Keywords:** semi-synthesis, opiate, alkaloid, antinociceptive, narcotic, natural product

## Abstract

Morphine and codeine, two of the most common opioids, are widely used in the clinic for different types of pain. Morphine is one of the most potent agonists for the *μ*-opioid receptor, leading to the strongest analgesic effect. However, due to their association with serious side effects such as respiratory depression, constriction, euphoria, and addiction, it is necessary for derivatives of morphine and codeine to be developed to overcome such drawbacks. The development of analgesics based on the opiate structure that can be safe, orally active, and non-addictive is one of the important fields in medicinal chemistry. Over the years, morphine and codeine have undergone many structural changes. The biological investigation of semi-synthetic derivatives of both morphine and codeine, especially morphine, shows that studies on these structures are still significant for the development of potent opioid antagonists and agonists. In this review, we summarize several decade-long attempts to synthesize new analogues of morphine and codeine. Our summary placed a focus on synthetic derivatives derived from ring A (positions 1, 2, and 3), ring C (position 6), and *N*-17 moiety.

## 1. Introduction

**Figure 1:**
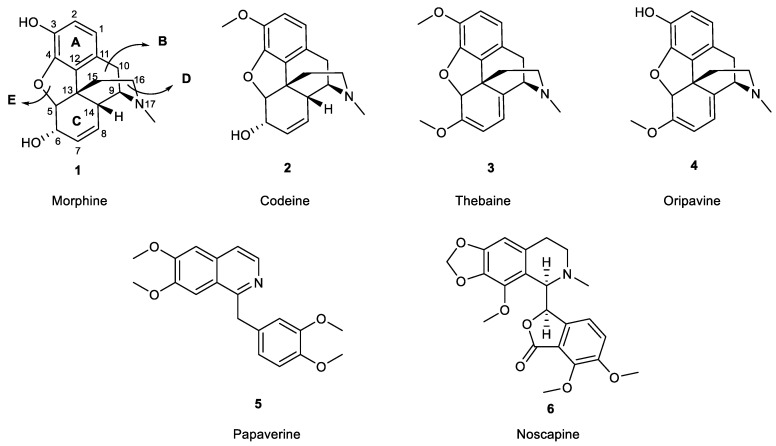
Structures of the most common opium alkaloids.

Natural products and their derivatives have historically played a significant role in drug discovery [1]. In 1805, the German chemist F. W. A. Sertürner isolated morphine from *Papaver somniferum* for the first time, which became a widely used medication to alleviate pain in the clinic [2]. In 1925, Robert Robinson proposed the chemical structure of morphine, and it was finally confirmed by X-ray crystallography in 1968. Codeine was isolated in 1832 by P. Robiquet [3]. Morphine is regarded as the “gold-standard” treatment for chronic and severe pain to which all other *μ*-agonists are compared. Morphine (4–20%) and codeine (0.7–5%) are the two major components of the opium poppy. Later, it was discovered that the latex of opium poppies contains other significant alkaloids, such as thebaine, papaverine, oripavine, and noscapine [4]. The structures of the most common alkaloids in opium poppy are depicted in the Figure 1.

As other phenanthrene alkaloids, morphine and codeine belong to the 4,5-epoxymorphinan class.

This review provides an overview of the analogues of morphine and codeine, in which the modification of the three main moieties, including ring A, ring C, and *N*-17, have been investigated. The biological effects of the most potent analogues are also mentioned.

Morphine, the main alkaloid in the mixture of over 20 alkaloids in opium, could act as the prototype *μ*-receptor agonist in the central nervous system (CNS), which is responsible for opium’s analgesic and sedative activities. Morphine activates three types of opioid receptors: the MOR (Mu Opioid Receptors, *μ*), KOR (Kappa Opioid Receptor, *κ*), and DOR (Delta Opioid Receptor, *δ*) receptors. They are all G-protein-coupled receptors (GPCRs) that activate G_i_ or G_o_ signal proteins [5,6]. The highest analgesic and sedative effects are produced when the *μ*-receptors are activated; however, this receptor is also associated with the strongest side effects. There are many side effects associated with morphine, such as respiratory depression, vomiting, constriction, nausea, euphoria, and addiction [7].

**Figure 2:**
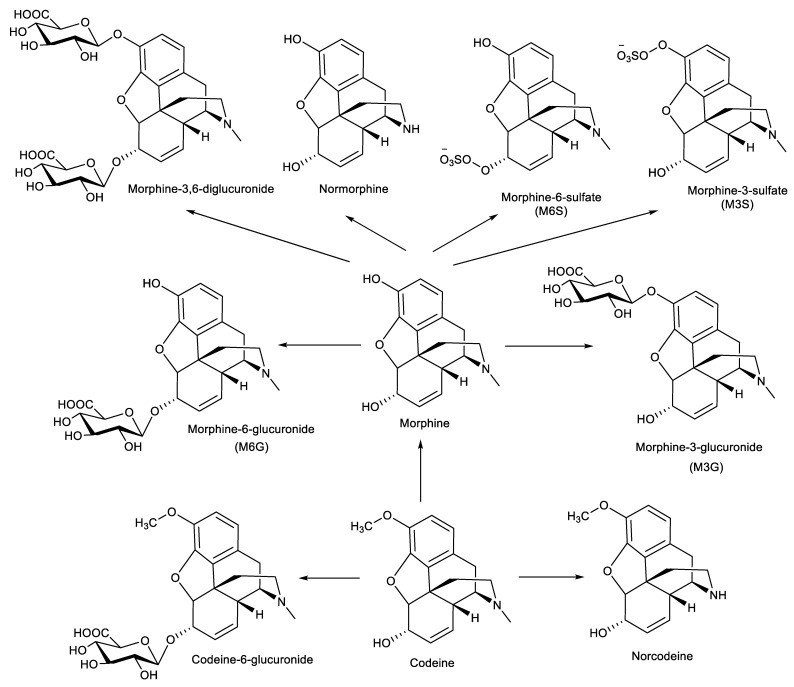
Metabolism of morphine and codeine.

Through phase II conjugation, morphine is mainly metabolized into morphine-3-glucuronide (M3G, 60%), morphine-6-*β*-glucuronide (M6G, 9%), and a smaller amount of *N*-demethylated metabolite (3%). Two uridine-diphospho-glucuronosyltransferase (UGT) enzymes are involved in morphine glucuronidation. Morphine is metabolized by UGT2B7 to both M3G and M6G, whereas UGT1A3 forms only MG3 [8]. Morphine-3-sulfate (M3S) and morphine-6-sulfate (M6S) have been detected in plasma of newborns. In general, during fetal life, sulfation is known as a significant pathway, while in adults, glucuronidation is much more important [9,10,11]. There are also some minor metabolism routes, such as diglucuronidation to morphine 3, 6-diglucuronide, and morphine ethereal sulfate (Figure 2) [12]. M3G has shown low affinity for *μ* receptors [13], while M6G, as a potent *μ* opioid receptor activator [14], has shown more analgesic activity than morphine with significantly less side effects [15,16]. Since morphine is relatively polar, it is not efficiently absorbed from the gut. Moreover, when administered intravenously, a vast percentage is blocked by the blood–brain barrier due to its quaternary ammonium salt form at physiological pH [17]. Prodrugs can be prepared by masking the polar functional groups of morphine, which allows them to cross the blood–brain barrier and enter the central nervous system more effectively [18]. Codeine, as a prodrug of morphine, has an analgesic effect about 10- to 12-fold lower than morphine and is prepared by methylation of the phenolic OH group. Most codeine is metabolized in the liver to its glucuronidated form and around 10% to morphine via *O*-demethylation by the CYP2D6 (CYP2D6 encodes the Cytochrome P450 family 2 subfamily D member 6 enzyme) pathway. There are also two other metabolites, codeine-6-glucuronide and norcodeine (Figure 2) [19,20,21]. 

The functional groups that are important for the binding interactions of morphine with the active site at an opioid receptor include the phenol OH group, which is essential for hydrogen bond formation, the protonated amine to form an ionic interaction, and the aromatic ring for the Van der Waals interaction with the hydrophobic position. There have been attempts to modify the structure of the opiate alkaloids in the past, and now several semi-synthetic derivatives of morphine and their congeners, such as naltrexone, naloxone, buprenorphine, and nalbuphine, are developed and used in the clinic as drugs.

## 2. Modifications on Ring A

### 2.1. Modifications on Position 1

Halogenation of C1 and C2 of morphine alkaloids has been noticed since the middle of the 20th century. Furthermore, halogenated morphinans have been known as valuable intermediates in the synthesis of tritium-labeled radioligands [22,23]. Halogenation at C1 of codeine was reported in 1925 by Speyer and Rosenfeld [24]. Hydrogen bromide was used in the presence of hydrogen peroxide at high temperatures to yield 1-bromocodeine **10**.

Singh et al. reported the synthesis of 1-chloromorphine (**7**) and 1-bromomorphine (**8**) in 1982 [25]. For the synthesis of **7** and **8**, morphine was treated with HCl or HBr solutions, respectively, in the presence of an oxidizing agent (KIO_3_ or H_2_O_2_). The results demonstrated that the bromination of morphine with this method directed bromine to the *meta* position of the phenolic hydroxyl group. We will discuss another method of directing halogen at the *ortho* position of morphine in the next section. 3-*O*-Methylation of **7** and **8** with diazomethane led to the formation of 1-chlorocodeine (**9**) and 1-bromocodeine (**10**), respectively. However, **7** and **8** can also be synthesized from the direct halogenation of codeine by the method explained above. 

**Scheme 1:**
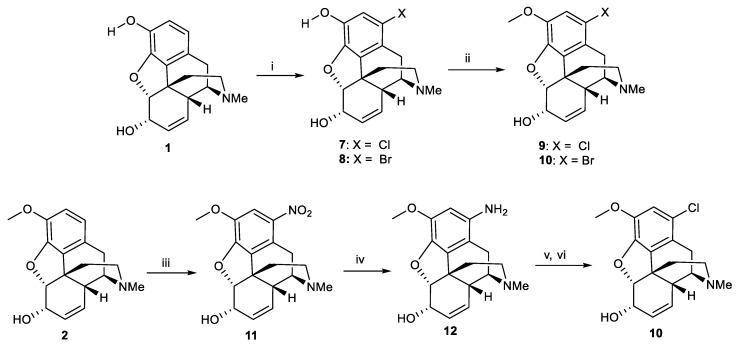
Reagents and conditions: (i) HX (Cl or Br), KIO_3,_ or H_2_O_2_; (ii) diazomethane, methanol/ether (1:1); (iii) HNO_3_, glacial acetic acid; (iv) SnCl_2_, HCl; (v) NaNO_2_, HCl; (vi) CuCl.

The same group investigated the synthesis of halogenated morphine and codeine from their corresponding nitro compounds, which will be discussed further in the following. Synthesis of the 1-aminocodeine **12** intermediate is an appropriate method to attach a variety of substituents in this position. Direct nitration of codeine [26] followed by reduction of the nitro group of **11** yielded 1-aminocodeine (**12**). Compound **12** underwent a Sandmeyer reaction to produce 1-chlorocodeine **10** (Scheme 1).

**Scheme 2:**
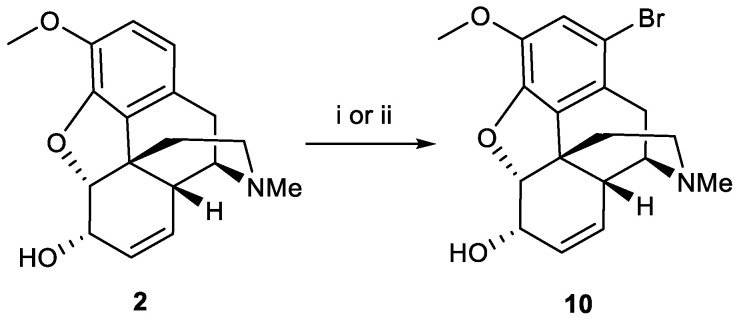
Reagents and conditions: (i) HBr, 30%, HCOOH, 30%. H_2_O_2_, 80 °C. 53%; (ii) NBA (1.08 eq.), TFAA (1.0 eq), THF, 0–15 °C, 24 h, 81%.

Moreover, a facile, safe, and yield-improving method has been developed for the synthesis of 1-bromocodeine **10** with *N*-bromoacetamide reagent in the presence of trifluoroacetic anhydride (Scheme 2) [27].

Braenden et al. reported the analgesic activity of the halogenated codeine analogues **9** and **10**, which demonstrated to have about 50% potency as compared to the parent compounds [28]. The hot plate test effectiveness of **8** was three times lower than morphine, but the potency of **7** was comparable to that of morphine [29]. Two hypotheses were considered to explain this decrease in pharmacological effect: the influence of steric effects on the proper attachment of the molecule to the receptor binding site and the effect of charge distribution on the aromatic ring. The first hypothesis was substantiated by the synthesis of 1-fluorocodeine (**16**) by Lousberg and Weiss in 1974 and the investigation of its biological properties in comparison with **9** and **10** [30]. Fluorine has been known as a prominent feature in drug design and development [31,32,33,34]. Replacement of hydrogen with fluorine can influence the pharmacokinetic properties of the molecules and affect their stability and bioavailability. According to the antinociceptive hot plate method, **16** had an ED_50_ of 7.9 mg/kg, and codeine **2** had an ED_50_ of 7.5 mg/kg. Comparison of other parameters of these two compounds did not show any significant difference neither in this method nor in the binding to the receptor of rat brain. In view of the mentioned results, since fluorine has a high electronegativity compared to chlorine and bromine, the second hypothesis was rejected. 

**Scheme 3:**
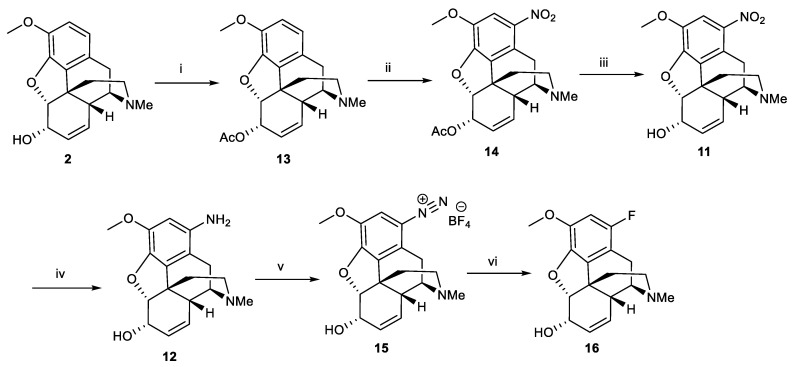
Reagents and conditions: (i) Ac_2_O, reflux; (ii) HNO_3_, glacial acetic acid, (1) 5 °C, 1 h, (2) room temperature, 1 h; (iii) 10% NaOH, ethanol, reflux, 1 h; (iv) SnCl_2_, HCl, r.t, 2 h; (v) 48% aqueous HBF_4_, EtOH, 2 M NaNO_2_, −15 °C to r.t; (vi) MgO, 170 °C, 1 h, in vacuo.

1-Fluorocodeine (**16**) was similarly synthesized from codeine via 1-aminocodeine in six steps [35]. First, codeine **2** was converted to 6-*O*-acetylcodeine (**13**) by reaction with acetic anhydride. Then, glacial acetic acid and nitric acid were added slowly to the solution of 6-*O*-acetylcodeine **13**. After the pH was adjusted to 9 with ammonia solution, **14** was precipitated. In this step, C6-ester was hydrolyzed with NaOH to obtain 1-nitrocodeine (**11**), which was then reduced to the corresponding amino derivative **12** with Stannous chloride/HCl. Treatment of **12** with tetrafluoroboric acid in anhydrous ethanol, followed by adding sodium nitrite solutions, led to the formation of diazonium salt **15** in 100% yield. In the last step, codeine-1-diazonium tetrafluoroborate salt **15** was heated in the presence of MgO based on the Makleit–Dubina method [36], which led to 1-fluorocodeine (**16**) (Scheme 3).

Aromatic iodides are chemically more reactive than chlorides and bromides. They are ideal intermediates for the synthesis of organometallic compounds or derivatization involving palladium-catalyzed reactions. In the past decades, several types of research have been conducted to synthesize novel 1-iodomorphine and codeine derivatives for nuclear medical imaging and radioimmunoassay [22,37,38].

**Scheme 4:**
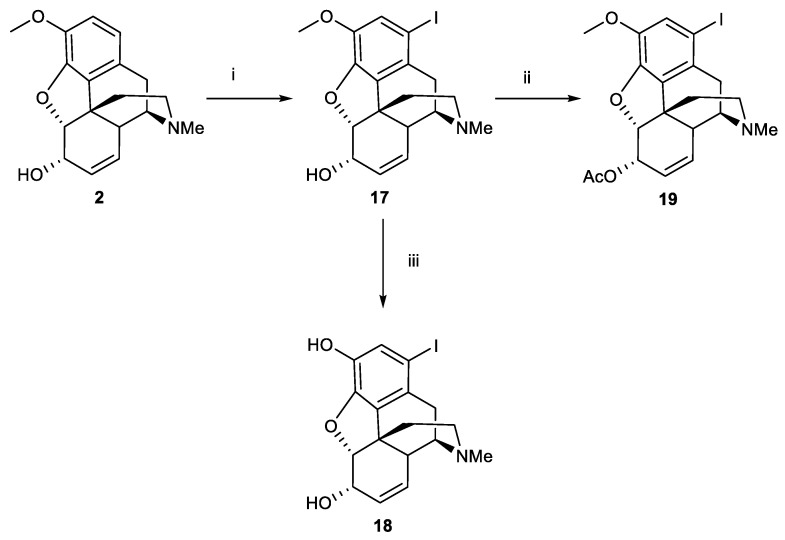
Reagents and conditions: (i) NaI, chloramine T, 0.1 M hydrochloric acid; (ii) Ac_2_O, reflux, 4 h; (iii) BBr_3_, CH_2_Cl_2_, r.t, 1 h.

In 1997, Liebman and co-workers reported the synthesis of 1-iodomorphine **18** and 1-iodocodeine **17** [39]. Aiming to obtain 1-iodocodeine (**17**), a solution of codeine **2** in HCl was added to sodium iodide and chloramine T. Under these conditions, no obvious reaction occurred with morphine. Therefore, 1-iodomorphine (**18**) was synthesized through an *O*-demethylation of codeine derivative **17** with BBr_3_. Compound 19 was obtained after acetylation of **17** with acetic anhydride, and its structure was confirmed by crystallography (Scheme 4).

**Scheme 5:**
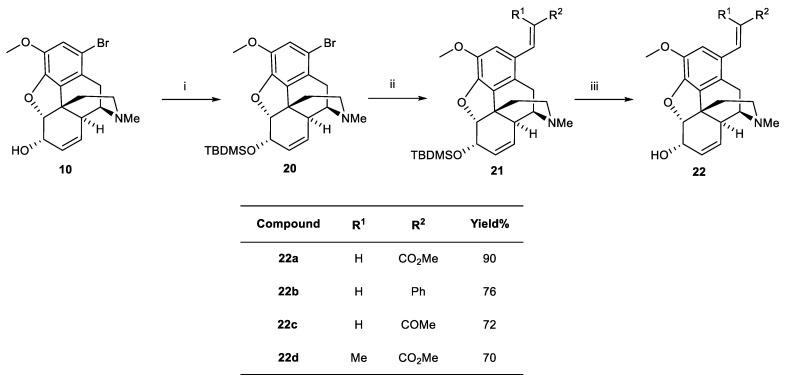
Reagents and conditions: (i) TBDMSCl (1.2 eq.), imidazole (1.2 eq.), DMF; (ii) Pd(OAc)_2_ (2 mol%), methyl acrylate (4 eq.), tri-*o*-tolylphosphine (8 mol%), NEt_3_ (1.4 eq.), DMF; (iii) TBAF (2 eq.), THF, r.t.

Several attempts were made to introduce different groups taking advantage of the halogen on 1-bromocodeine **10** via palladium-catalyzed coupling reactions. Davies et al. synthesized novel 1-vinyl, -acyl, and -alkyl codeine derivatives through Heck-type reactions [40]. To prevent any side reactions, the allylic hydroxyl was protected by *tert*-butyldimethylsilyl chloride to yield **20**. Treatment of **20** with a variety of vinyl compounds in the presence of Pd(OAc)_2_, phosphine ligand, and NEt_3_ led to the formation of 1-vinylcodeine derivatives. Methyl acrylate, styrene, methyl vinyl ketone, and methyl methacrylate were used as vinyl substrates, followed by deprotection of the silyl group of **21** with TBAF, which led to the formation of **22a**–**d**, respectively. These derivatives **22a**–**d** were identified as a single diastereoisomer with (*E*)-geometry at the double bond via ^1^H-NMR analysis based on the coupling constants (Scheme 5).

**Scheme 6:**
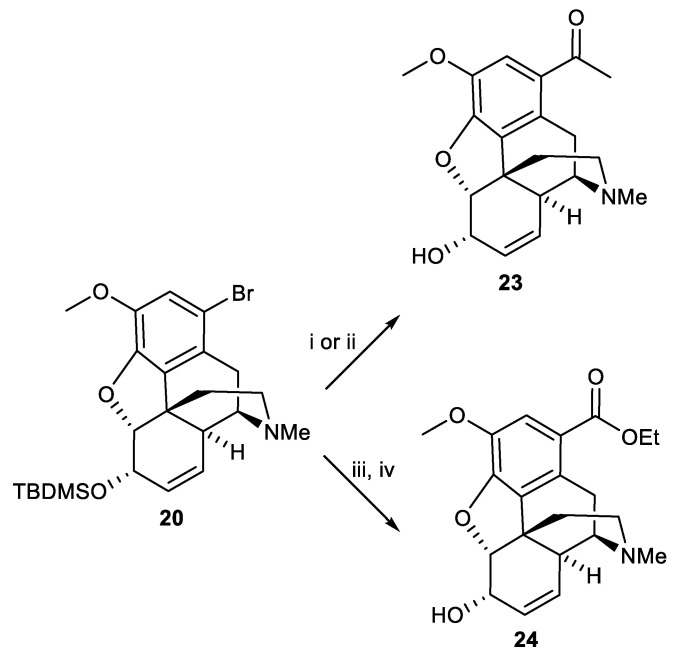
Reagents and conditions: (i) Pd(OAc)_2_ (2 mol%), ethyl vinyl ether (4 eq.), tri-*o*-tolylphosphine (8 mol%), NEt_3_ (1.4 eq.), DMF, then HCl (aq), 39%; (ii) Pd(OAc)_2_ (2 mol%), (*α*-ethoxyvinyl)tributyltin (4 eq.), PPh_3_ (8 mol%), NEt_3_ (1.4 eq.), DMF, then HCl, 85% (aq); (iii) PdCl_2_ (2 mol%), PPh_3_ (8 mol%), CO (3 atm), NEt_3_–EtOH; (iv) TBAF (2 eq.), THF.

In the second approach, 1-acetylcodeine (**23**) was synthesized with a similar methodology. The synthesis procedure was performed by the involvement of either ethyl vinyl ether or (*α*-ethoxyvinyl)tributyltin in different types of palladium-catalyzed reactions. The results showed that the use of the Stille-type coupling with (*α*-ethoxyvinyl)tributyltin compared to the Heck process with ethyl vinyl ether increased the reaction yield from 39% to 85% (Scheme 6).

To better investigate the reactivity of compound **20**, 1-carboethoxy and 1-alkyl derivatives were synthesized as well. 1-Ethoxycarbonylcodeine (**24**) was obtained in 50% yield by carbonylation in the presence of carbon monoxide followed by silyl deprotection. Palladium (II) chloride was used as a source of palladium in this reaction (Scheme 6).

**Scheme 7:**
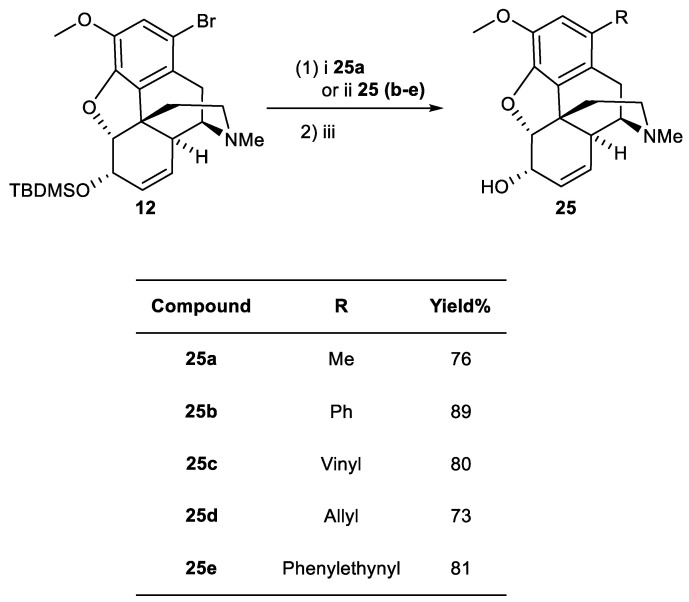
Reagents and conditions: (i) Pd(OAc)_2_ (2 mol%), tri-*o*-tolylphosphine (8 mol%), Me_4_Sn (1.2 eq.), NEt_3_ (1.4 eq.), DMF; (ii) Pd(OAc)_2_ (2 mol%), PPh_3_ (8 mol%), Bu_3_SnR (1.2 eq.), NEt_3_; (iii) TBAF (2 eq.), THF, r.t.

By the same authors, the production of 1-alkylation of codeine by a modified Stille procedure was also attempted [41]. Therefore, the methyl derivative **25a** was synthesized by reacting **12** with tetramethyltin in the presence of Pd(OAc)_2_. In the following, some differently substituted R-tributyltins were used to produce four other 1-substituted codeine derivatives **25b**–**e** in good yields (Scheme 7). Part of this work was conducted by Davies and Pyatt in 1989 [42].

1-Aminocodeine (**12**) is a key intermediate towards synthesizing several azo, thiazole, and hydrazine derivatives which have structures that are attractive probes in medicinal chemistry.

**Scheme 8:**
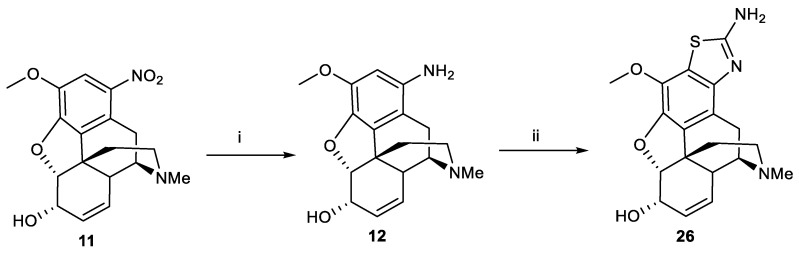
Reagents and conditions: (i) FSA, NaOH; (ii) KSCN, Br_2_, AcOH, 60 W, 130 °C, 15 min.

In 2008, Sipos et al. reported the synthesis of A-ring-fused 2-aminothiazole from **12** [43]. As a starting point, codeine was again converted to **11** [44]. For the reduction of the nitro analogue **11** to the corresponding amino compound **12**, formamidinesulfinic acid (FSA) was used instead of the older method of using hydrogen sulfide. An attempt to synthesize the compound **26** using bromine with potassium thiocyanate in acetic acid (Kaufman method) was associated with low yield [45]. The possibility of radical intermediates in such reactions led the scientists to apply high temperature and microwave (MW) induction as successful optimizations (Scheme 8), and a mechanism was proposed for this reaction with radical intermediates, supported by DFT (Density Functional Theory) calculations.

**Scheme 9:**
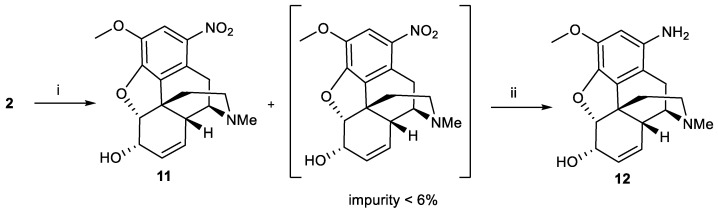
Reagents and conditions: (i) Bi(NO_3_)_3_·5H_2_O, CH_3_COOH, r.t, 6 h, 69%; (ii) SnCl_2_, 35% HCl, r.t, 2 h, 86%.

**Scheme 10:**
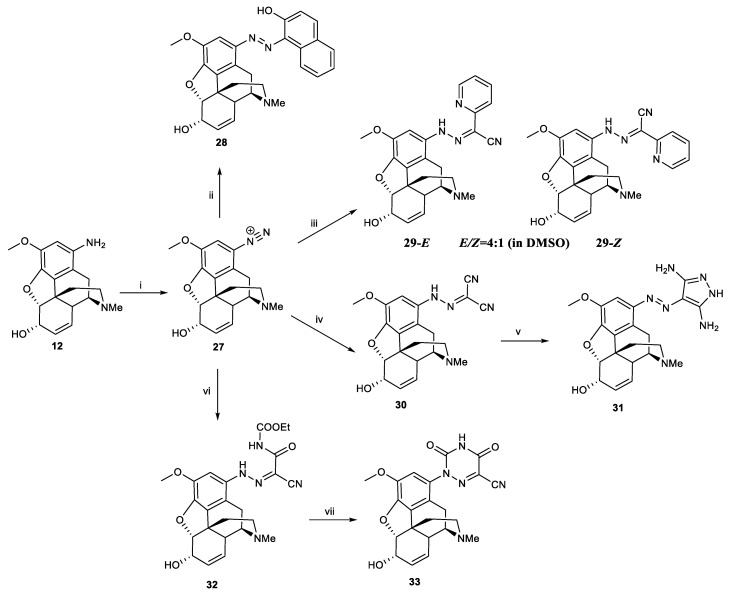
Reagents and conditions: (i) NaNO_2_, HCl, NaBF_4_, H_2_O, 5–7 °C, 15 min; (ii) 2-Naphthol, NaOH, H_2_O, 5–7 °C, 18 h, 97%; (iii) 2-(pyridin-2-yl)acetonitrile, CH_3_COONa, H_2_O, 5–7 °C, 18 h, 67%; (iv) malononitrile, CH_3_COONa, H_2_O, 3–5 °C, 3 h; (v) N_2_H_4_·H_2_O, MeOH, 65 °C, 3 h, 48% (over two steps); (vi) ethyl (2-cyanoacetyl)carbamate, NaOH, H_2_O, 3–5 °C, 1 h; (vii) 47% (over two steps).

With the aim to synthesize azo and hydrazone derivatives of codeine, Cankar et al. used **11** as a starting material [46]. Initially, a novel nitration process was developed using bismuth nitrate pentahydrate (instead of nitric acid) as a mild nitrating agent with a 69% yield. Then, the nitro group was reduced to an amino group (Scheme 9). In this study, the structures of **11** and **12** were confirmed by X-ray analysis. Diazonium salt **27** was synthesized by the reaction of **12** with sodium tetrafluoroborate. 2-Pyridylacetonitrile and 2-naphthol were reacted with **27** in the presence of a base to produce a mixture of *E*/*Z* isomer **29** (67%) and the *E* isomer of **28** (97%), respectively (Scheme 10). In the subsequent steps, intending to synthesize heterocyclic derivatives, compound **27** underwent reaction with malononitrile to afford hydrazone **30**, followed by reaction of **30** with hydrazine to yield diaminopyrazole **31**. Another analogue was obtained through a coupling reaction of **27** with (2-cyanoacetyl)carbamate, which cyclized directly to 6-azauracil **33** (Scheme 10). 

### 2.2. Modifications on Position 2

**Scheme 11:**
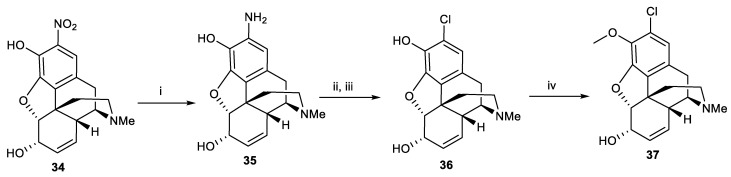
Reagents and conditions: (i) Sn/HCl, FSA, or electrolytic reduction; (ii) HCl, NaNO_2_; (iii) CuCl; (iv) ethereal diazomethane, MeOH.

Halogenation at position 2 was achieved by converting morphine (**1**) to 2-aminomorphine (**35**) [25,44]. Initially, 2-nitromorphine (**34**) was prepared with the nitration being regioselective for position 2 due to the presence of the free hydroxyl group at the 3-position. Tin and hydrochloric acid [44], electrolytic reduction [44], and reduction utilizing formamidinesulfinic acid [44] were the three main methods for the reduction of nitro derivatives to the corresponding amino analogues. 2-Chloromorphine (**36**) was prepared through the Sandmeyer reaction from **35**. Furthermore, treatment of **36** with ethereal diazomethane in methanol led to the formation of 2-chlorocodeine (**37**) (Scheme 11) [24].

**Scheme 12:**
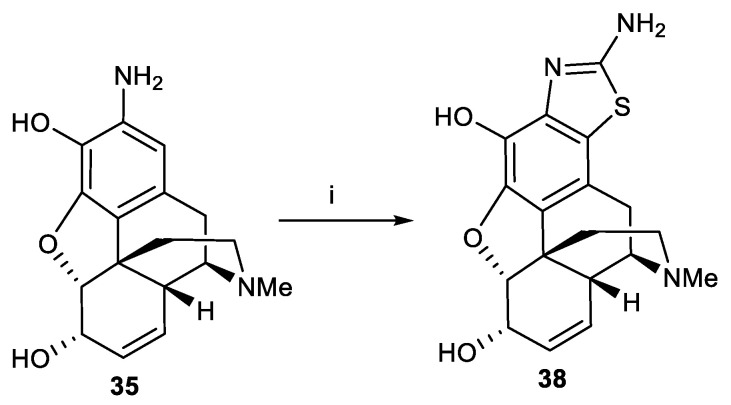
Reagents and conditions: (i) KSCN, Br_2_, AcOH, 60 W, 130 °C, 15 min.

The formation of the 2-aminothiazole ring (**26**) from 1-nitrocodeine (**12**) has already been explained in the previous section. The same group demonstrated that a similar methodology can be applied to the synthesis of ring A-fused 2-aminothiazole **38** starting from 2-nitromorphine (**35**) (Scheme 12) [43]. 

### 2.3. Modifications on Position 3

According to several studies, the 3-hydroxy group in morphine is necessary to form a hydrogen bond in its target binding site. Researchers have studied the interaction of these derivatives with opioid receptors over the years by substituting different groups in this position.

The palladium-catalyzed reaction was used to synthesize new C3-substituted derivatives of morphine [47]. As expected, substitution at the C3 position showed no improvement in morphine potency at any opioid receptors. However, the selectivity of 3-furyl analogue **40b** at *μ*-receptors was close to that of morphine. 

**Scheme 13:**
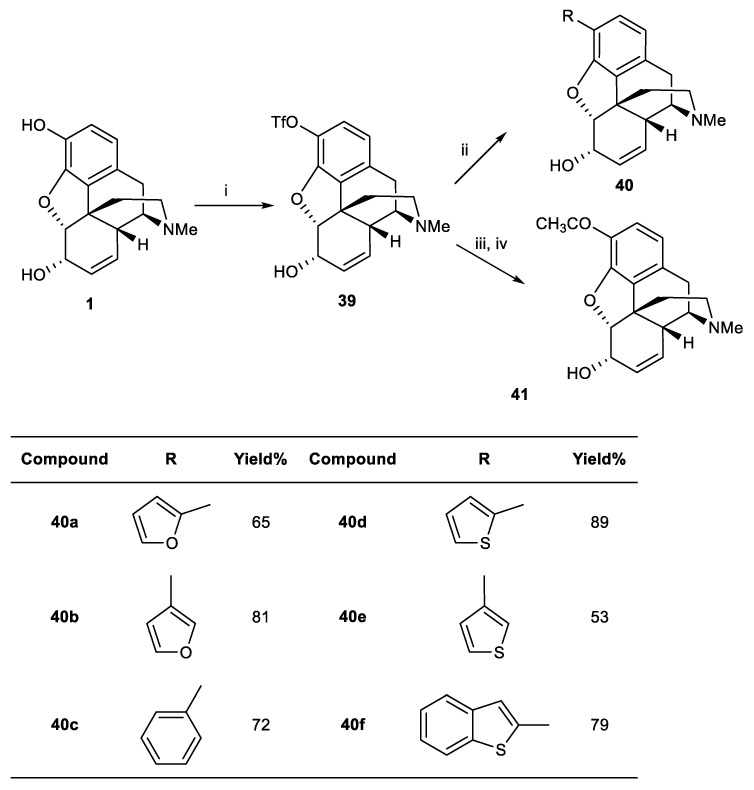
Reagents and conditions: (i) *N*-phenyltrifluoromethanesulfonimide, CH_2_Cl_2_; (ii) RB(OH)_2_, Pd(PPh_3_)_4_, LiCl, 2M Na_2_CO_3_, EtOH, DME; (iii) CH_2_=C(OC_2_H_5_)-SnBu_3_, PPh_3_, PdCl_2_(PPh_3_)_2_, DMF, 2,6-di-*t*-butyl-4-methylphenol; (iv) THF, 1 M HCl.

Treatment of morphine with *N*-phenyltrifluoromethanesulfonimide and triethylamine led to the formation of triflate derivative **39**. Palladium-catalyzed coupling reaction of **39** with (1-ethoxy)vinyltributyltin and hydrolysis resulted in methylketone **41**. By the Suzuki–Miyaura reaction of the different arylboronic acids with **39** in the presence of palladium-tetrakis(triphenylphosphine), C3-aryl-substituted analogues **40a**–**f** were obtained in 53–89% yields (Scheme 13). The affinity of novel derivatives for opioid receptors was lower than morphine; however, the selectivity of compound **40b** was comparable to that of morphine (Table 1).

Previous work of Wentland and co-workers on cyclazocine analogues [48] had shown that mono *N*-substitution on the 8-OH group was found to have an affinity for opioid receptors, and it seems that replacing phenolic OH by NH can enhance the binding affinity. It can be demonstrated that NH_2_ can play an isostere role for the 3-OH of morphine for opioid receptors. 

The 3-OH group of morphine can be replaced by the amino group using the Pd-catalyzed amination Buchwald methodology starting from aryl triflates [49]. Wentland et al. reported new morphine analogues based on this method [50]. In the first step, 3,6-hydroxy groups were both protected to form 3,6-bis-*tert*-butyldiphenylsilyl ether **42** through the reaction of morphine (**1**) with imidazole and excess *tert*-butyldiphenylsilyl chloride (TBDPSCl). To produce the monoprotected 6-*tert*-butyldiphenylsilyl ether **43**, **42** was reacted with tetrabutylammonium fluoride. The best results for high selectivity 3-TBDPS deprotection of compound **42** were obtained with 0.25 eq of TBAF (1 M in THF containing 5% H_2_O). In this reaction, water may play a crucial role in catalyzing the reaction by converting the HF (resulting from hydrolyzing TBDPSF produced by 3-TPDPS cleavage) to TBAF, which can desilylate another **42** on the catalytic cyclic. Compound **43** was converted to the 3-triflate **44** using a standard method.

**Scheme 14:**
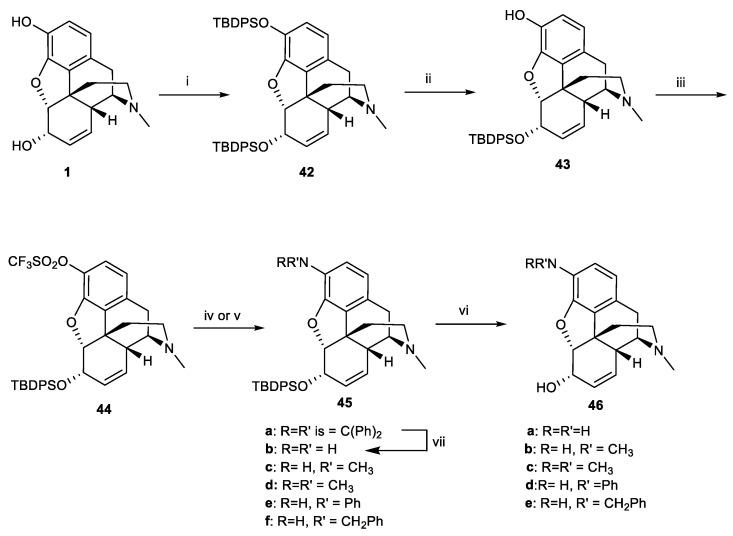
Reagents and conditions: (i) TBDPSCl, imidazole, CH_2_Cl_2_, 25 °C, 1 h, quant; (ii) 0.25 eq. TBAF, THF/H_2_O, 25 °C, 1 h, 84%; (iii) *Tf*_2_O, pyr, CH_2_Cl_2_, 0 °C, 1 h; (iv) RR’NH, *t*-BuONa, toluene, 80 °C, Pd(OAc)_2_, BINAP; (v) Pd_2_(dba)_3_, DPPF, 26–84%; (vi) 1.5 eq.TBAF, THF/H_2_O, 25 °C, 6–12 h, 79–96%; (vii) NH_2_OH.HCl/NaOAc, MeOH, 97%.

Two known Pd-catalyzed amination reactions were used for synthesizing derivatives **45c**–**f** [49]. In one of them, the combination of Pd(OAc)_2_ with BINAP was used, and in the other method, Pd_2_(dba)_3_ with DPPF were used. Hydrolysis in the last step gave the desired derivatives **46b**–**e** in 79–96% yields. To obtain compound **46a**, **45a** was converted to **45b** under imine exchange conditions, followed by deprotection of the 6-hydroxyl group (Scheme 14).

Subsequently, binding affinity and selectivity of the synthesized derivatives **46a**–**e** on *μ*, *δ*, and *κ* opioid receptors in guinea pig membranes were investigated in comparison to morphine. All derivatives showed significant decrease in affinity for *µ* (38–273-fold), *δ* (11–41-fold), and *κ* (10–141-fold) opioid receptors. As a consequence of substituting hydrogen in **46a** with methyl (**46c**), the affinity for *μ* receptors decreased, although no significant difference was observed between **46c** and **46d**. The analogue **46e** showed the highest *μ* affinity and highest *µ:δ* and *µ:κ* selectivity ratios in the series (Table 2). The results showed that replacement of the 3-OH group of morphine with -NH_2_ as a bioisoster generally had a favorable effect on the activity. 

**Scheme 15:**
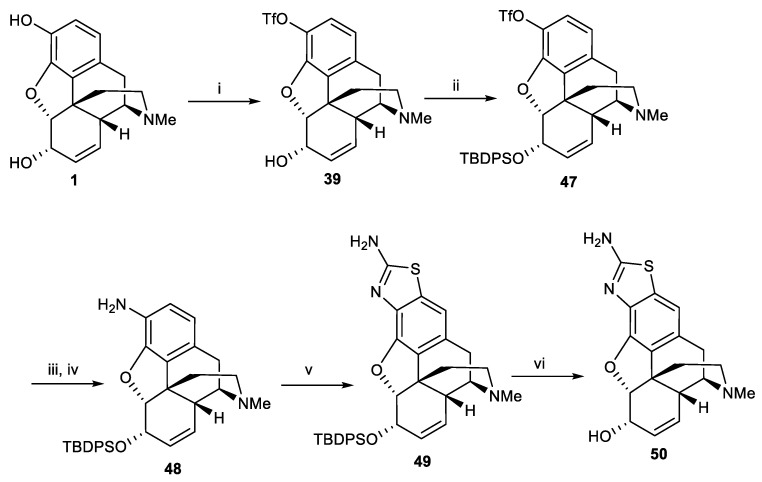
Reagents and conditions: (i) PhN*Tf*_2_, Et_3_N; (ii) TBDMSCl, imidazole, THF; (iii) Pd(OAc)_2_, BINAP, Ph_2_C=NH, Cs_2_CO_3_; (iv) NaOAc, NH_2_OH.HCl; (v) KSCN, Br_2_, AcOH, 23.4%; (vi) TBAF, THF, 62.2%.

The A-ring-fused 2-aminothiazole derivative **50** was reported as a bioisostere of phenolic hydroxyl [51]. Morphine **1** was reacted with PhN*Tf*_2_ to selectively convert the phenolic hydroxyl group to the triflate **39**. After protection of the allylic hydroxyl with TBDPS, compound **47** was obtained. The next amination step to achieve **48** was the same as the method reported by Wentland et al. [50]. Treatment of **48** with KSCN and Br_2_, followed by deprotection of allylic hydroxyl group, led to the formation of **50** (Scheme 15). This heterocyclic bioisostere, aminothiazole, has been successfully applied to dopamine agonists, for instance, B-HT 920 [52] and PD 118440 [53], to improve their pharmacological properties. In comparison to the high affinity of morphine **1** at the *µ* receptors, this aminothiazole derivative (**50**) displayed a 34-fold (K_i_ = 30 nM) and a 9-fold (K_i_ =220 nM) decrease in affinity against *μ* and *κ* receptors, respectively, and a 4-fold (K_i_ = 32 nM) increase in affinity at *δ* receptors compared to the phenol, levorphanol (K_i_ (*μ*) = 0.21 nM, K_i_ (*δ*) = 4.2 nM, K_i_ (*κ*) =2.3 nM). Compound **50** also showed a 7-fold higher affinity at *κ* receptors than morphine (K_i_ = 24 nM), and at *μ* and *δ* receptors, no significant difference was observed (Table 3).

**Scheme 16:**
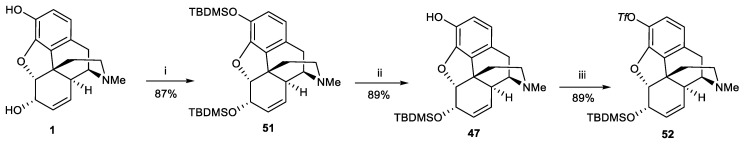
Reagents and conditions: (i) TBDMSCl (3 eq.), imidazole (5.0 eq.), THF; (ii) TBAF (1 eq.), THF, 0 °C; (iii) trifuoromethanesulfonic anhydride (1.2 eq.), 2,6-dimethylpyridine (1 eq.), DCM, 0 °C.

**Scheme 17:**
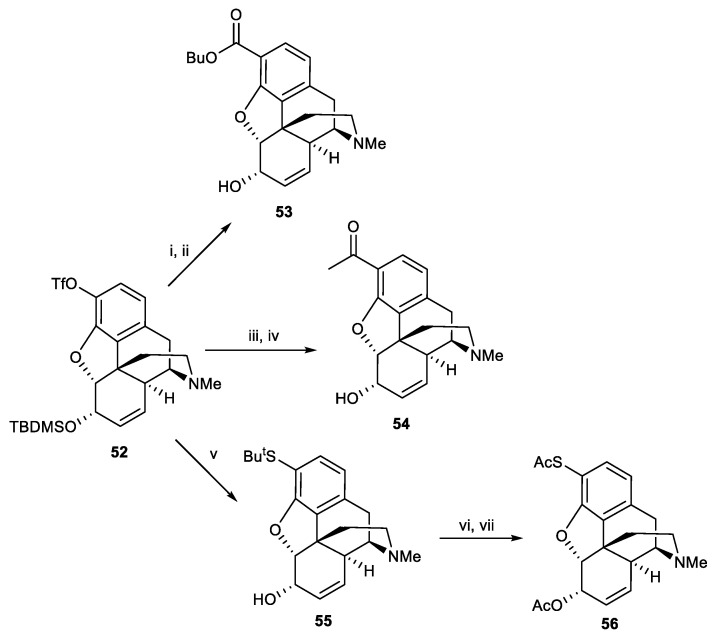
Reagents and conditions: (i) CO (3 atm), n-BuOH, Pd(OAc)_2_ (4 mol%), 1,3-bis(diphenylphosphino)propane (4 mol%), heat (ii) TBAF, THF, 76% (overall two step); (iii) (α-ethoxyvinyl)tributylstannane, Pd(OAc)_2_ (4 mol%), PPh_3_ (8 mol%), LiCl, DMF, heat; (iv) HCl(aq), r.t, 80% (overall two steps); (v) Pd(PPh_3_)_4_ (0.5 eq.), tributylstannyl *tert*-butyl sulfide (2 eq.), LiCl (3 eq.), DMF, 83%; (vi) Hg(OAc)_2_ (1.05 eq.), anisole, TFA, 0 °C, then H_2_S; (vii) acetic anhydride, NEt_3_, DMAP, DCM, 47% (overall two step).

The synthesis of some derivatives in position 1 of codeine through the palladium-catalyzed reactions was mentioned in the previous section. The same group used this reaction to synthesize new 3-morphine analogues [40]. Initially, compound **51** was obtained in 87% yield from the protection of two hydroxyl groups of morphine with *tert*-butyldimethylsilyl chloride. Towards **52**, first the phenolic group of 3,6-di-*O*-*tert*-butyldimethylsilylmorphine **51** was selectivity deprotected by TBAF to yield **47**, followed by conversion to triflate **52** (Scheme 16). Compounds **53** and **54** were synthesized from **52** in the presence of Pd(OAc)_2_ through the Stille and Pd-catalyzed carobonylation reactions, respectively. Substitution of sulfur at the 3-position resulted in the formation of **56.** Triflate **52** was reacted with tributylstannyl *tert*-butyl sulfide and Pd(PPh_3_)_4_ to produce **55**. The *tert*-butyl group deprotected with mercuric acetate and TFA and then treated with hydrogen sulfide gas and acetic anhydride led to the formation of **56** in 47% overall yield (Scheme 17).

**Figure 3:**
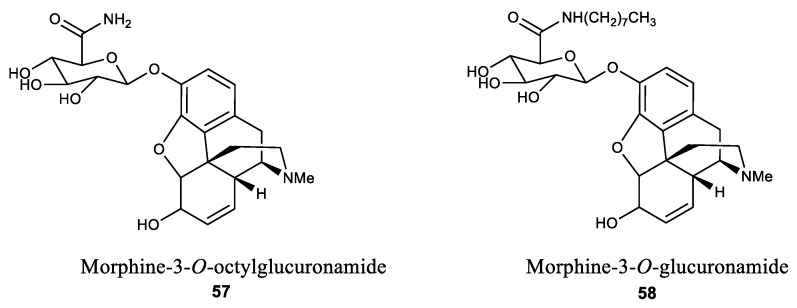
Structures of morphine-3-*O*-octylglucuronamide and morphine-3-*O*-glucuronamide.

In 2012, Salvatella et al. synthesized two lipophilic analogues of M3G by forming an amide bond between a primary alkylamine and carboxylic acid group of M3G [54]. The synthesis procedures were explained in detail in the review of sugar derivatives of morphine by Jash and Gorai [55]. A surprising result of biological activity in rats and mice showed that increasing lipophilicity by an aliphatic octyl chain produced highly toxic derivative **58**. The opioid antagonist activity of **57** showed the same properties as naloxone without being toxic (Figure 3).

## 3. Modifications on Position 6

**Scheme 18:**
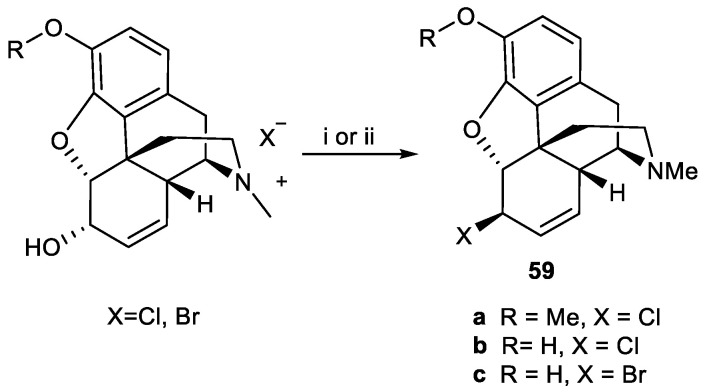
Reagents and conditions. (i) Dimethylchloroformiminium chloride, DMF; (ii) PPh_3_, diethyl azodicarboxylate (DEAD), or diisopropyl azodicarboxylate (DIAD), benzene or toluene, r.t, 1 h.

*α*-Halogenated-codides and -morphides are known to be key intermediates for substituting with nucleophiles at this allylic position. *α*-Chlorocodide (59a) was synthesized through the reaction of codeine **2** with thionyl chloride by the method of Wieland and Kappelmeier [56]. Stork and Clarke showed that the *α*-chlorocodide reacts with nucleophiles such as piperidine and thioethanol by an S_N_2 mechanism [56]. In 1976, the synthesis of *α*-chloromorphine 59b from the reaction of morphine hydrate with dimethylchloroformiminium chloride (Vilsmeier reagent) was reported (Scheme 18) [57]. The measurement of the analgesic potency and toxicity has shown that *α*-chloromorphine was about 10–15 times more potent than morphine, but in vivo test showed it was significantly more toxic, again compared to morphine.

Compounds **59a**–**c** were also synthesized via the Mitsunobu reaction [29,58]. The hydrochloride salts of codeine and morphine were reacted with azocarboxylates (DEAD or DIAD) in the presence of triphenylphosphine. It was shown that the hydrogen halide produced in situ in this reaction can act as an acid component. The reaction proceeded with chloride and bromide with an inversion of configuration at the C6 position to obtain **59a**–**c** (Scheme 18).

**Scheme 19:**
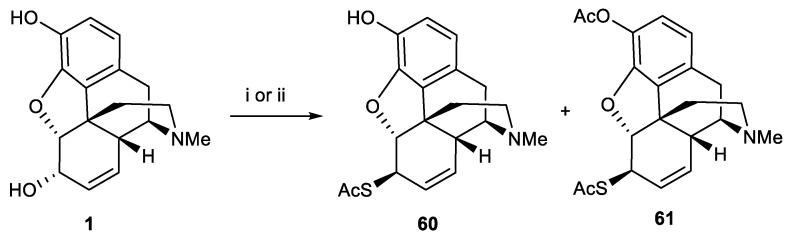
Reagents and conditions: (i) AcSH, *N*,*N*-dimethylformamide dineopentyl acetal, dry toluene, 80 °C; (ii) AcSH, 2-nitrobenzenesulfenyl chloride, PPh_3_, dry THF, 0 °C.

**Scheme 20:**
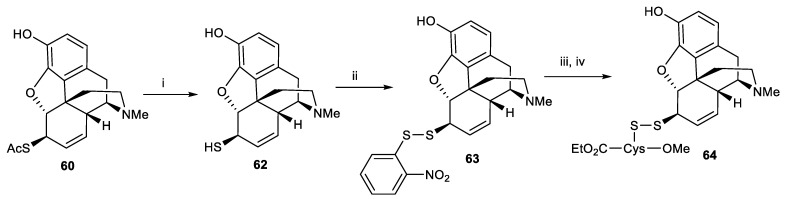
Reagents and conditions: (i) 0.2 N KOH, EtOH, N_2_ (96%); (ii) 2-nitrobenzenesulfenyl chloride, CH_3_CN, 0 °C (78%); (iii) L-Cys-OMe.HCl, EtOH, or DMF; (iv) ethyl chloroformate (3 eq), K_2_CO_3_ (anh), CH_3_Cl, 0 °C.

In 1988, Fujii et al. reported the novel analogues of 6-*β*-thiomorphine [59]. The C6-hydroxyl group was replaced with the thiol group. Two independent procedures were examined to synthesize compounds **60** and **61**. In the first step, morphine was treated with thioacetic acid and *N*,*N*-dimethylformamide dineopentyl acetal. This reaction resulted in the formation of **61** as the major product. In the second procedure, morphine underwent the Mitsunobu reaction with thioacetic acid and triphenylphosphine (PPh_3_) in the presence of diisopropyl azodicarboxylate (DIAD). In this reaction compound **60** was prepared in 73% yield as the major product (Scheme 19). The stereochemistry of **60** and **61** at the C6 position was assigned as the *β*-orientation based on ^1^H-NMR spectroscopy. Compound **63** underwent the reaction with L-cysteine methyl ester followed by ethyl chloroformate and K_2_CO_3_, producing carbamate **64** (Scheme 20). It has been demonstrated that this sulfhydryl morphine derivative **63** binds to the μ-receptors from the disulfide bond [60]. Furthermore, the analgesic activity of compound **60** was five times higher than morphine in rats.

By converting the C6-hydroxyl of morphinans to amine, a group of compounds called aminomorphinans have been produced. The synthesis of pharmacologically active analogues of aminomorphinans through the Mitsunobu reaction led to the development of a variety of derivatives [61,62,63]. 

The Mitsunobu reaction has gained much attention in the field of natural products [64,65,66]. Several researchers have reported the application of this method for the synthesis of novel morphine derivatives.

**Scheme 21:**
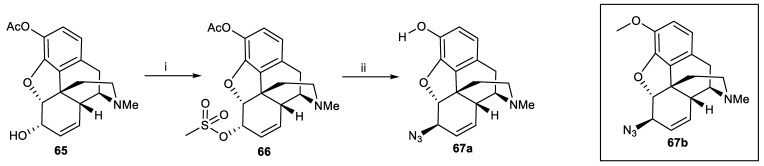
Reagents and conditions: (i) mesyl chloride, pyridine, 47.5%; (ii) aq.sodium azide, DMF, 24 h, 100 °C, 50.3%.

In 1968, Makleit and Bognár reported the new derivative of morphine and codeine that obtained much attention in the following years [67]. 6-Deoxy-6-azido-dihydroisomorphine (azidomorphine **67a**) and d 6-deoxy-6-azido-dihydroisocodeine (azidocodeine **67b**) were synthesized. It was demonstrated that azidomorphine has 40 to 300 times, and azidocodeine has about 13 times, more potency than morphine in animal [68] and human [69] clinical trial studies. The ED_50_ of **67a** and **67b** in the tail flick and hot plate test were reported in Table 4 [70]. It has been demonstrated that the incidence of side effects was significantly decreased in patients who were treated with azidomorphine. The reaction of **65** with methanesulfonyl chloride and pyridine yielded compound **66**. Treatment of mesylates **66** with sodium azide through an S_N_2 reaction with inversion of configuration followed by removal of the 3-acetyl group led to the formation of azidomorphine **67a** (Scheme 21) [71]. The dependence liability and tolerance of azidomorphine in mice and rats were much lower than that of morphine [72].

**Scheme 22:**
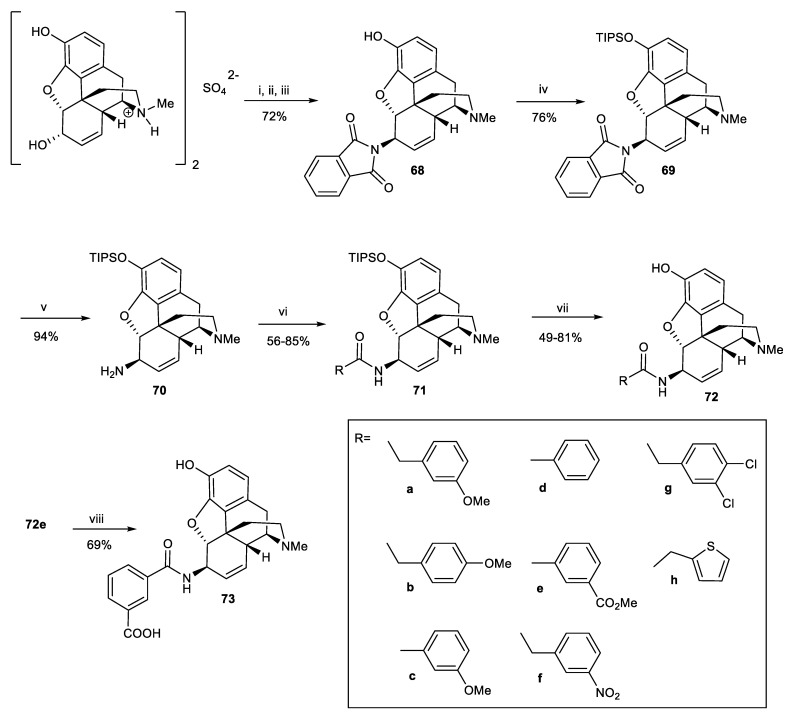
Reagents and conditions: (i) Ac_2_O, NaHCO_3_, H_2_O; (ii) PPh_3_, *i*-PrO_2_CN=NCO_2_*i*-Pr, phthalimide; (iii) NH_2_OH.HCl, EtOH, 55 °C; (iv) TIPSCl, imidazole, DMF; (v) NH_2_NH_2_, EtOH, 55 °C; (vi) RCOCl, NEt_3_, CH_2_Cl_2_; (vii) TBAF, THF; (viii) LiOH.

In 2004, MacDougall and co-workers attempted to synthesize novel 6-arylamidomorphine derivatives as morphine-6-glucuronide (M6G) analogues by introducing different aryl groups instead of saccharides [73]. First, the 6-*α*-hydroxyl group was converted to 6-*β*-phthalimide **68** through the Mitsunobu reaction. The phenolic hydroxyl group of morphine was selectively acetylated, and the resultant allylic alcohol subsequently reacted with DIAD and phthalimide in the presence of Ph_3_P. This was followed by the removal of the C-3 acetyl group, affording compound **68**. Treatment of 6-*β*-phthalimidomorphine **68** with imidazole and triisopropylsilyl chloride (TIPSCl) followed by deprotection of the phthalimide group in the presence of hydrazine hydrate led to the formation of primary amine **70**. The amide derivatives **71a**–**h** were obtained from the reaction of **70** with a variety of arylmethyl and heteroarylmethyl acid chlorides. Deprotection with TBAF produced the desired 6-*β*-amidomorphines **72a**–**h**. Subsequently, hydrolysis of **72e** with lithium hydroxide gave the isophthalic acid derivative (**73**) (Scheme 22).

Analogues **72a**–**h** exhibited 21–64-fold higher affinity for *μ* receptors compared to M6G and 1.9–5.5-fold higher affinity than morphine. The affinity of compound **73** for the *μ* receptors was significantly lower than other derivatives. The [^35^S]GTPγS assay was used to evaluate the potency or affinity of derivatives for the opioid receptors. [^35^S]GTPγS binding was maximal with full agonists and reduced with partial agonists. Based on the EC_50_ values, all derivatives showed full *μ* agonist effects except **72b** and **73,** which were partial *μ* agonists. Compound **73** was chosen for the tail flick test in rats, and it showed weak antinociceptive activity with ED_50_ = 12.6 mg/kg at 60 min. These results showed well the pharmacological effects of substituting arylamide groups with saccharide portion in M6G. Part of these biological data are summarized in Table 5.

**Scheme 23:**
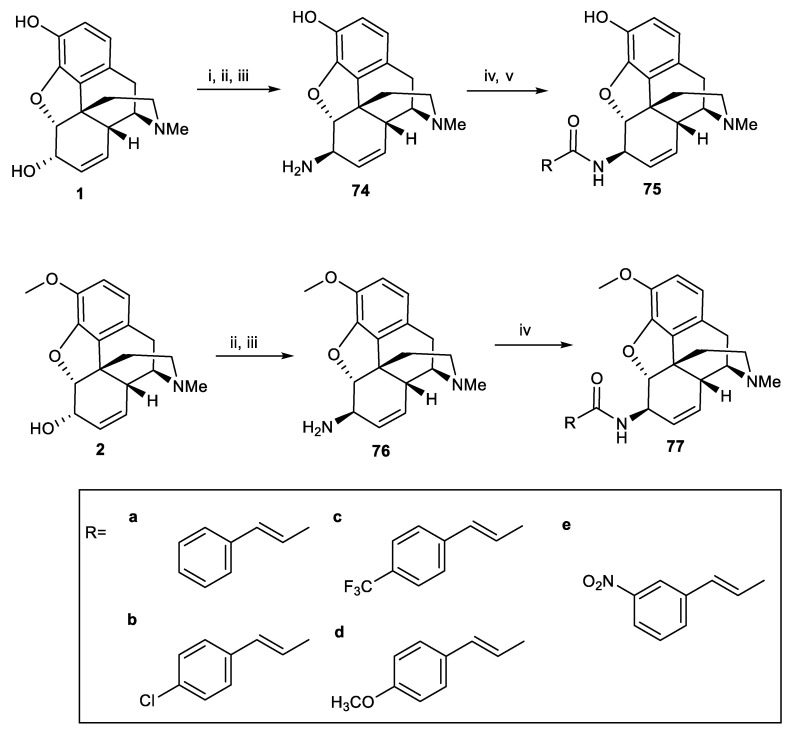
Reagents and conditions: (i) acetic anhydride, NaHCO_3_, H_2_O, r.t, 1 h; (ii) phthalimide, DIAD, Ph_3_P, benzene, r.t, 2h; (iii) hydrazine hydrate, EtOH; (iv) 1.1 eq. thionyl chloride, RCOOH, Et_3_N, CH_2_Cl_2_, r.t, 2 h; (v) Na_2_CO_3_, H_2_O, MeOH, r.t, 12 h.

Following the efforts to synthesize new analogues of M6G in 2013, Váradi et al. synthesized novel 6*β*-acylamides of morphine **1** and codeine **2** [74]. Initially, 6*β*-amino derivatives **74** and **76** were synthesized using the Mitsunobu reaction. Treatment of 6*β*-amino morphine or codeine in NEt_3_ with thionyl chloride and appropriate carboxylic acids led to the formation of the desired **75a**–**e** and **77a**–**e** derivatives, respectively (Scheme 23). 

In vitro binding studies showed that cinnamoyl morphinamines had a moderate to high affinity for MOR-1 (Table 6). In contrast, the affinity of the corresponding codeine derivatives was significantly lower than the cinnamoyl morphinamine analogues. The analgesic potency of **75a** (ED_50_ = 3.13 ± 1.09 mg/kg) as the most effective analogue was similar to that of morphine (ED_50 =_ 4.96 ± 0.97 mg/kg) but without causing respiratory depression, which has been the main side effect of traditional opiates. The [^35^S]GTPγS binding assay for the most potent derivative (**75a**) showed that it acts as a full agonist for all opioid receptors (MOR-1 agonist DAMGO, KOR-1 agonist U50,488H, and DOR-1 agonist DPDPE). 

**Figure 4:**
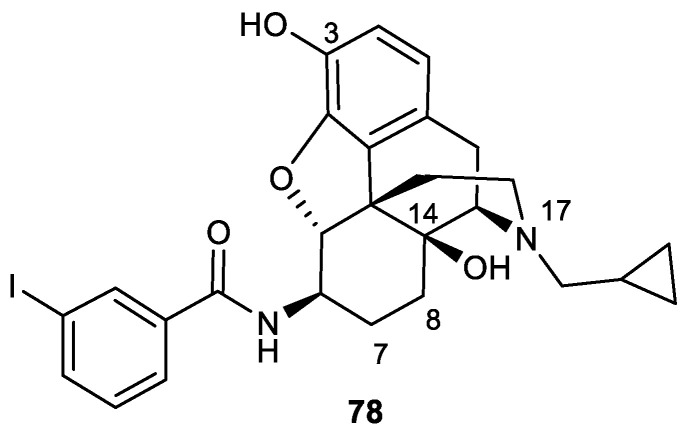
Structure of 3-iodobenzoyl naltrexamine.

**Scheme 24:**
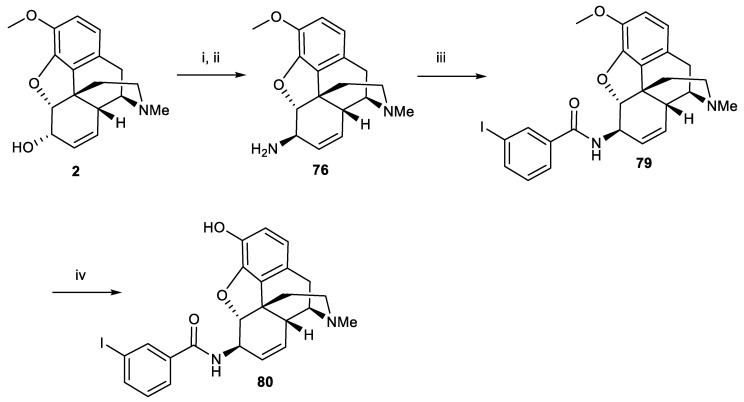
Reagents and conditions: (i) phthalimide, Ph_3_P, dry THF, DIAD in dry toluene, 16 h, r.t, 93%; (ii) hydrazine hydrate, *cis*-2-penten-1-ol, MeOH, 16 h, r.t, 100%; (iii) 3-iodobenzoic acid, 1-[bis(dimethylamino)methylene]-1*H*-1,2,3-triazolo[4,5-*b*]pyridinium 3-oxide hexafluorophosphate (HATU), *N*,*N*-diisopropylethylamine (DIEA), dimethyl formamide (DMF), 15 min, r.t, 76%; (iv) BBr_3_, DCM, 30 min, 0 °C to r.t, 55%.

In 2015, the same group synthesized five analogues of 3-iodobenzoyl naltrexamine (IBNtxA) **78**, a potent aminomorphinan antinociceptive drug (ED_50_ = 0.39 mg/kg) [75]. However, it had a lower affinity for the target site, 6TM/E11, compared to traditional opioid receptors. The 6-transmembrane (6-TM) is a novel opioid target that is known as a novel exon 11-associated splice variant of the μ-opioid receptor gene. An attempt was made to modify these structures by removing the 14-hydroxy group, substituting different alkyls at the N-17 position, and adding a double bond at positions 7–8 (Figure 4). Derivatives were synthesized starting from codeine. First, 6*β*-aminocodeine **76** was synthesized from **2**, and the amine derivative was subsequently coupled with 3-iodobenzoic acid in the presence of HATU to produce compound 79. *O*-Demethylation of compound **79** with BBr_3_ led to the formation of 80 (Scheme 24).

The affinity of compound **79** for 6TM/E11 and DOR-1 was moderate, whereas it showed high affinity for MOR-1 (1.2 nM) and KOR-1 (10 nM) receptors. Analogue **80** demonstrated a high affinity for all opioid receptors (Table 7). This analogue, in contrast with the corresponding 14-OH-epoxymorphinan, exhibited moderate affinity (27 nM) for 6TM/E11 and analgesic potency in vivo. Although incorporating a double bond at the 7–8-positions increased the affinity to MOR, KOR, and DOR, it reduced the selectivity to the 6TM/E11 site. The modification of the N-17 position will be explained in the next section.

**Figure 5:**
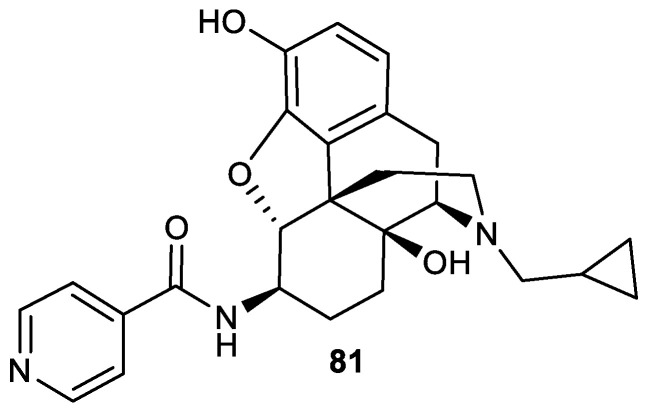
Structure of compound **81**.

After synthesizing a very potent and selective *μ* opioid receptor (compound **81**) (Figure 5) derived from **80** in 2009 [76], it was selected as a lead compound for further study. In 2017, a novel series of 6*β*-heteroarylamidomorphinanes was synthesized to modify some parts of **81** to investigate structure–activity relationship of this potent analogue [71].

**Scheme 25:**
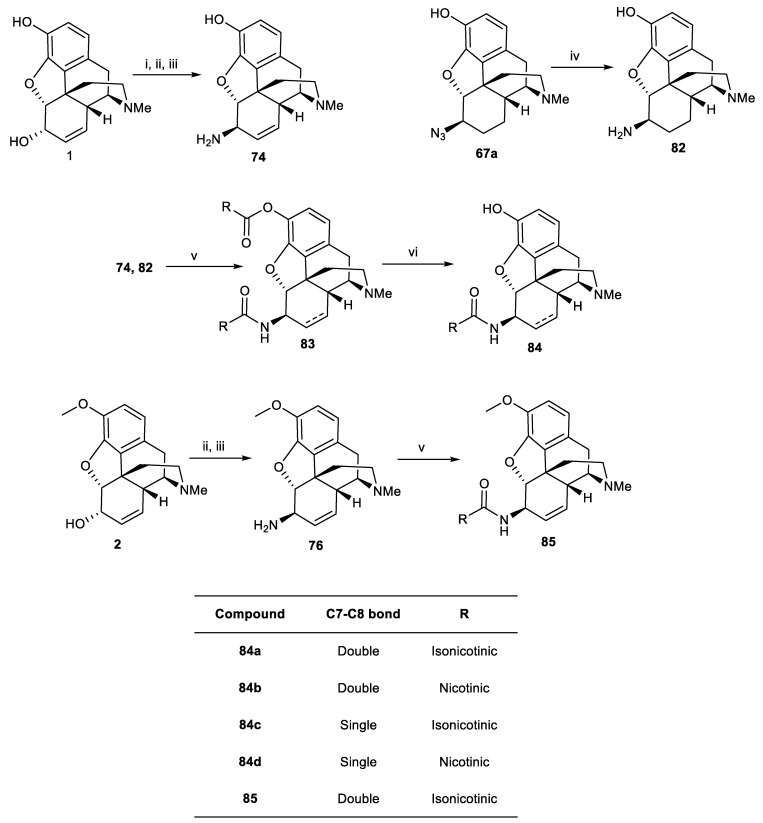
Reagents and conditions: (i) acetic anhydride, aq. NaHCO_3_, r.t, 2 h; (ii) Ph_3_P, phthalimide, DIAD, benzene, r.t, 1 h; (iii) ethanol/hydrazine monohydrate, heating, 3 h; (iv) hydrazine monohydrate/RANEY nickel, ethanol, r.t., 2 h; (v) acyl chloride, Et_3_N, DCM; (vi) K_2_CO_3_, methanol, heating, 4 h.

Treatment of 6β-aminomorphine **74** or the corresponding dihydromorphine analogue **82** with isonicotinic and nicotinic acid chlorides led to the formation of the 6*β*-aminomorphinans **84a**–**d**, respectively. A similar reaction was performed between 6*β*-aminocodeine **76** and isonicotinic acid chloride to produce **85** (Scheme 25).

Based on in vitro studies, the codeine analogue **85** showed significantly lower binding affinity compared to morphine derivatives **84a**–**d**. This illustrated the importance of the free hydroxyl group at the C-3 position. Derivatives **84a** and **84b** revealed the highest affinity to the MOR compared with the analogues bearing 14-hydroxyl group and a saturated C7-C8. Based on the [^35^S]GTPγS binding assay of compounds **84a**–**d**, all derivatives were full agonists, and only the ED_50_ value for **84d** was determined. The [^35^S]GTPγS assay is summarized in Table 8. Saturating the double bonds in these derivatives decreased both affinity and activity, despite the literature indicating that saturation of double bonds at positions 7–8 increased morphine derivative affinity. According to the tail flick test, **84b** (ED_50_ = 0.51 μg) was as potent as morphine (ED_50_ = 0.53 μg). The **84c** and **84d** with saturated double bonds, on the other hand, exhibited long-lasting analgesic effects.

As mentioned before, M6G is one of the metabolites of morphine in the body. This potent analgesic is four times more active than, and twice as long-lasting, as morphine. Due to the low bioavailability of M6G, scientists are interested in developing new glycosylated derivatives of morphine at the C6 position. Many attempts have been made to synthesize new C6-glycosylated morphine, and most of them were collected in the earlier review of S. K. J. Dilip Gorai [55]. Here, we have only mentioned some more potent analogues.

**Figure 6:**
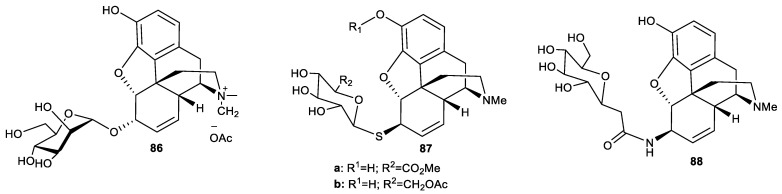
Structure of potent C6-glycosylated derivatives.

6-Morphinyl-R-*D*-mannopyranoside **86** (Figure 6) is one of the potent analogues which was synthesized by Arsequell and co-workers, which was twice as long-lasting when administered intraperitoneally to rats and was found to have a 100-fold naloxone-reversible antinociceptive action [77].

Due to the low bioavailability of M6G and the fact that the *S*-glycoside linkage has more in vivo stability in comparison with *O*-glycosides in metabolic conjugates, MacDougall et al. synthesized novel 6-*β*-thiosaccharide derivatives of morphine and codeine [78]. Compounds **87a** and **87b** (Figure 6) were determined as full *μ* agonists and showed a slightly better potency (around 1.5-fold) on *μ* receptors compared to M6G in the [^35^S]GTPγS assay. Based on the tail flick test, the ED_50_ of **87a** was 2.5 mg/kg, which was slightly lower than morphine (3 mg/kg). Due to the low selectivity of these derivatives for *μ* in comparison with *δ* and *κ* receptors, the same group synthesized two new amide-linked C-*β*-glycopyranoside derivatives of M6G [79]. It was found that compound **88** had a 3.7-fold greater affinity for *μ* receptors than M6G and had selectivity ratios of 76.7 and 166 for *δ* versus *μ* and *κ* versus *μ* receptors, respectively (Figure 6). Compound **88** also showed high stability at pH 2 and pH 7.4.

**Scheme 26:**
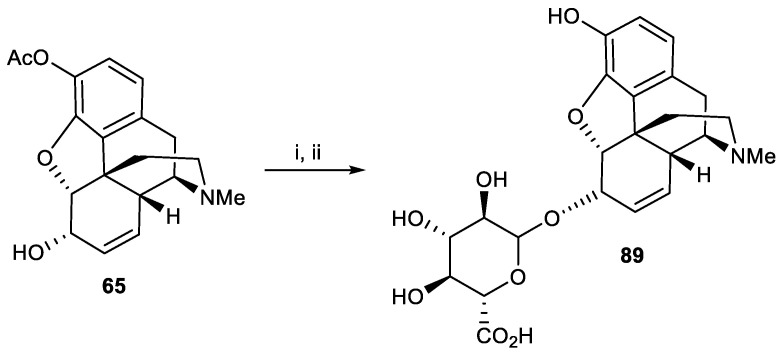
Reagents and conditions; (i) methyl 2,3,4-tri-*O*-acetyl-D-glucopyranosyluronate bromide, ZnBr_2_, molecular sieves, CHCl_3_; (ii) NaOH 5%.

In 2020, Yang et al. synthesized the M6*α*G analogue and evaluated its biological activity compared to its isomer M6*β*G (M6G), an active metabolite of morphine [80]. M6*α*G was synthesized from the reaction of 3-*O*-acetylmorphine with methyl 2,3,4-tri-*O*-acetyl-D-glucopyranosyluronate bromide and ZnBr_2_ followed by hydrolyzing the acetyl group in 5% NaOH (Scheme 26). In the first step, a mixture of two isomers (*α*:*β* = 4:1 by HPLC) was obtained. After treating the mixture with hydrochloric acid, the M6*α*G **89** was crystallized in hot water. Both of these analogues demonstrated a nanomolar affinity for all *μ*, *δ*, and *κ* opioid receptors. However, in the hot plate test, compound M6*α*G showed lower analgesic activity than M6G.

## 4. Modifications at *N*-17

In many alkaloids, particularly opiates, the tertiary *N*-methylamine group is a characteristic moiety. The removal and substitution of the *N*-methyl group in the natural products, especially in the alkaloids, is considered a significant step in the processes of semi-synthesis for further functionalization and modification to produce novel compounds with improved pharmacological and biological activities [81]. The methyl group in position 17 of the morphine scaffold is one of the most common sites for modification, and many derivatives of morphine and other structurally related analogues have been developed via the replacement of this group with other substituents. A wide variety of procedures for the *N*-demethylation of opiate alkaloids have been reported in the literature. The more conventional methods involve the use of reagents like cyanogen bromide (von Braun reaction), chloroformate esters, and dialkyl azodicarboxylates. The toxicity of cyanogen bromide is one of the major disadvantages of the von Braun reaction, which significantly limits the use of this method both in the laboratory and in the industry [81,82]. Recently, the modified non-classical Polonovski reaction has been employed for the *N*-demethylation of opiates in good to high yields through the formation of the corresponding *N*-oxide and its subsequent treatment with FeSO_4_.7H_2_O to afford the *N*-nor secondary amine opiates [83]. Furthermore, various efficient photochemical and biochemical methods have been successfully employed for *N*-demethylation of opiate alkaloids [84,85].

The methyl group in position 17 of the morphine scaffold is one of the most common sites for substitution and modification, and many derivatives of morphine and other structurally related analogues have been developed via the replacement of this group with other substituents.

**Figure 7:**
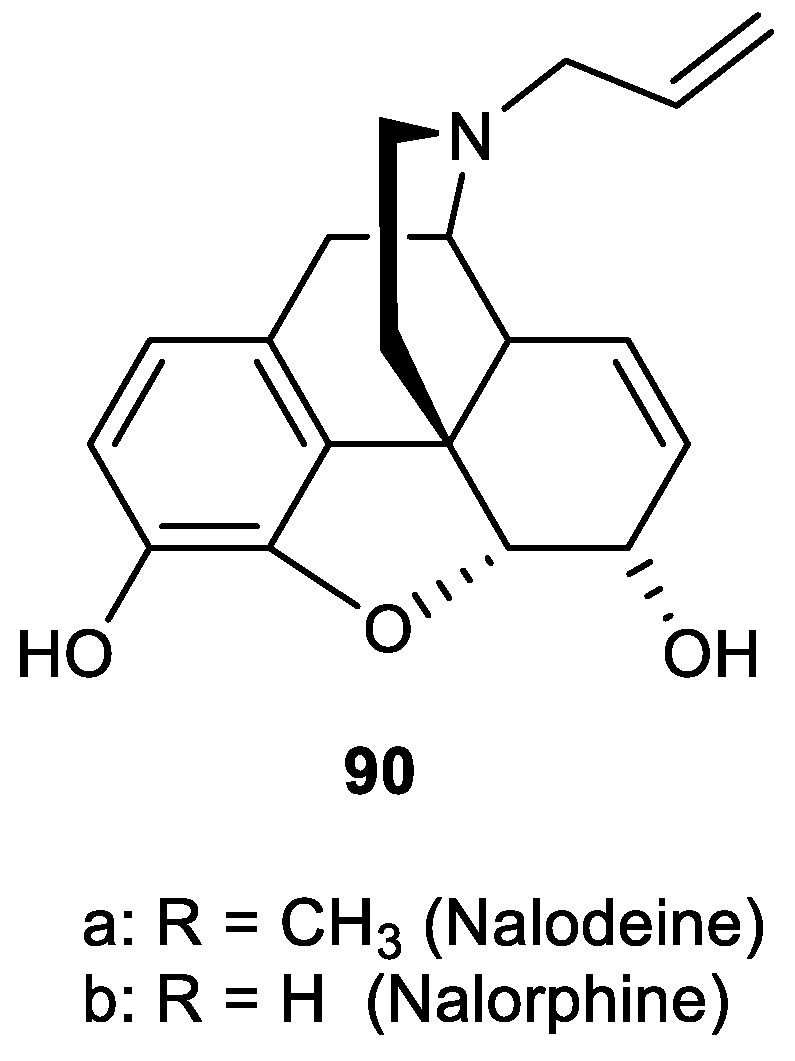
Structures of nalorphine and nalodeine.

*N*-Allylnorcodeine (nalodeine) **90a** and *N*-allylnormorphine (nalorphine) **90b** are notable as the first *N*-substituted analogues of morphine and codeine (Figure 7) [86]. It was demonstrated that nalorphine does not have analgesic activity in animals. In the writhing test, nalorphine-6-glucuronide and -sulfate showed higher analgesic activity that nalorphine. Nalorphine has both agonist and antagonist properties [87].

In the following years, many researchers synthesized hundreds of *N*-substituted normorphines to better understand their structure–activity relationship. Here, we have summarized some of these synthesized derivatives and, in some cases, their biological properties.

In 1953, Clark and co-workers synthesized several *N*-substituted normorphine **91a**–**r** and dihydromorphine **92a**–**f** analogues [88]. Some of these derivatives are summarized in Table 9 and Table 10. It was found that substitution of allyl, methallyl, *n*-propyl, or isobutyl in all series produced compounds that counteracted morphine’s analgesic effects.

**Figure 8:**
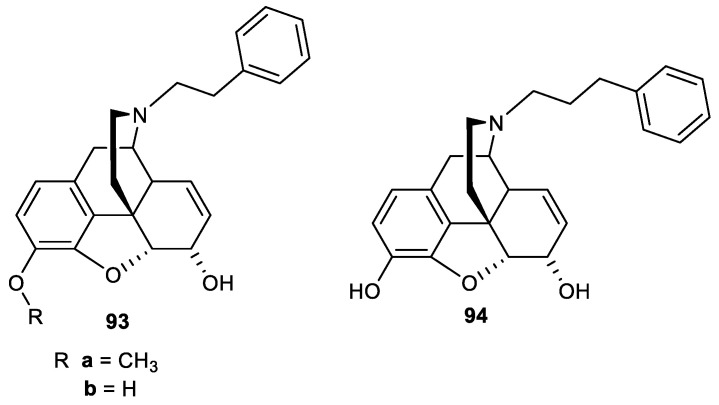
*N*-phenethyl derivative of morphine.

In 1958, Small et al. synthesized *N*-phenethylnorcodeine [89]. Initially, norcodeine was reacted with phenylacetyl chloride, and then the resultant amide was reduced to the tertiary amine using lithium aluminum hydride to give the desired product **93a**. *N*-Phenethylnormorphine **93b** was synthesized in the same manner (Figure 8). Investigation of the analgesic potency of **93a** showed that it provided analgesic action that was twice as long as its *N*-methyl counterpart.

Ben Haddou et al. synthesized *N*-phenylpropylnormorphine (**94**) and compared the opioid receptor binding and antinociceptive activity of this analogue with *N*-phenethylnormorphine (**93b**) [90]. It was demonstrated that **94** has high affinity and selectivity at the *μ* opioid receptors (MOR) as a potent agonist (K_i_ = 0.93 nM) and displayed an affinity for the MOR that was seven times higher than morphine. Compound **94** had significantly reduced binding affinity and selectivity at μ receptors (Table 11).

**Scheme 27:**
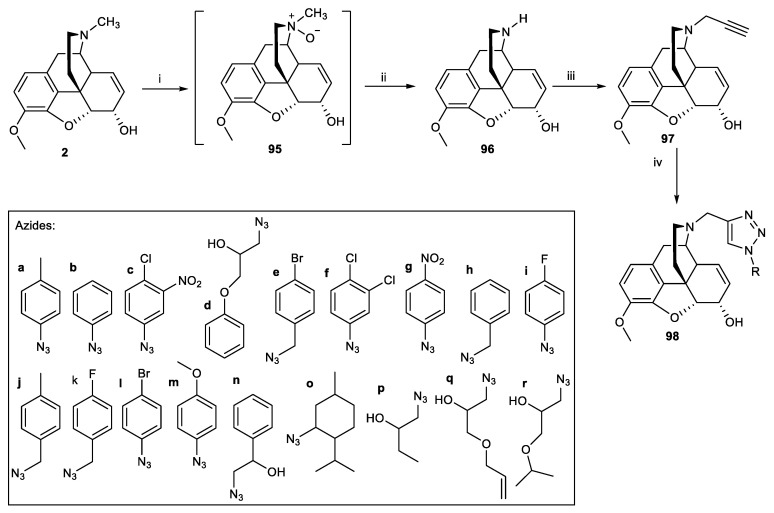
Reagents and conditions: (i) H_2_O_2_, acetonitrile, r.t, 4 h; (ii) FeSO_4_.7H_2_O, MeOH, −5–0 °C, 8h, 57%; (iii) propargyl bromide, K_2_CO_3_, acetone, r.t, 4 h, 86%; (iv) R-N_3_, CuSO_4_, sodium ascorbate, MeOH/DCM (1:1), r.t, 10–60 min.

In 2020, Salehi et al. synthesized a new series of triazole-tethered derivatives of norcodeine [91]. *N*-Demethylation of codeine (**96**) was successfully carried out with a good yield via the Polonowski-type reaction in the presence of hydrogen peroxide and FeSO_4_.7H_2_O. Then, **96** was treated with propargyl bromide to produce *N*-propargylnorcodeine (**97**). From the CuAAC reaction of **97** with a variety of azides, the desired triazole derivatives **98a**–**r** were prepared (Scheme 27). Radioligand Binding Assay showed that derivatives have an affinity to *μ* receptor. Compound **98d** (K_i_ =159.9 nM; IC_50_ =363.1 nM) showed high affinity, and compound **98k** (K_i_ = 850.6 nM; IC_50_ = 1931.0 nM) showed the lowest affinity compared to codeine (K_i_ = 144.2 nM; IC_50_ =327.4 nM). Compounds **98a**, **98c**, **98d**, **98f**, **98h**, and **98r** with high affinity were selected for tail flick analgesic test. The results showed that Compound **98d** exhibited significant analgesic activity with an ED_50_ of 8.68 mg/kg compared to codeine (ED_50_: 9.55 mg/kg) as the best analogue (Table 12).

## 5. Disubstituted Derivatives

### 5.1. Modifications on C3–C6

Previous results have demonstrated that glucuronidation is the main metabolism pathway of morphine, whereas less attention has been devoted to the sulfation pathway. According to the tail flick assay, M6S showed a 30-fold higher analgesic potency than morphine, similar to morphine-6*β*-glucuronide (M6G) [92,93]. Codeine-6-sulfate (C6S) showed similar potency ratios to that of M6S, whereas a high toxicity level for C6S was exhibited [94]. This issue grabbed the attention of scientists and led to the synthesis of various sulfated derivatives.

**Scheme 28:**
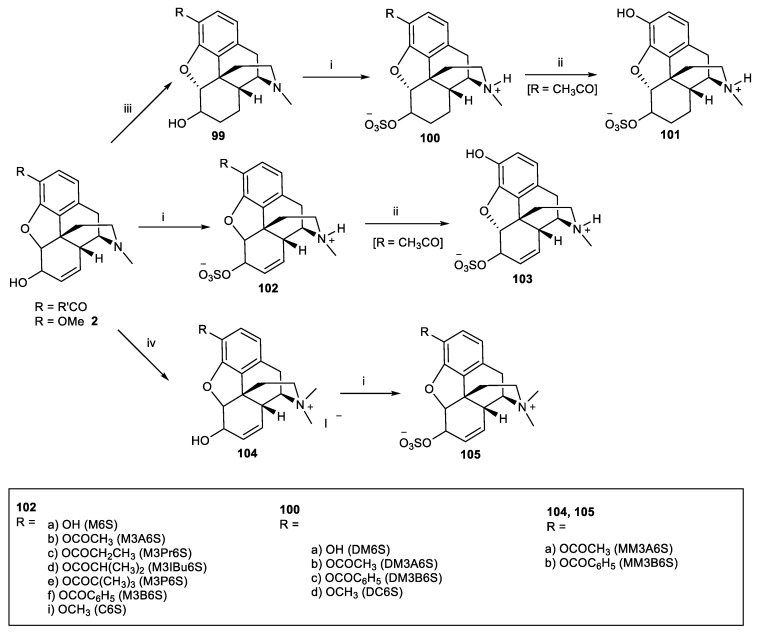
Reagents and conditions: (i) pyridine-SO_3_, pyridine, 55 °C; (ii) 5% MeOH, aq. NaOH, r.t, 1 h; (iii) Pd-C, 5%, H_2_, 50psig; (iv) CH_3_I/CH_2_Cl_2_, r.t, 12–18 h.

In 2006, Crooks et al. reported the synthesis of 6-*O*-sulfate ester derivatives starting from codeine and morphine and their corresponding dihydro analogues to investigate their binding affinity to several opioid receptors [95]. A variety of 3-*O*-acyl-6-*O*-sulfate derivatives were synthesized by treating codeine and 3-*O*-acyl analogues of morphine with pyridine-SO_3_ to produce **102a**–**i**. The dihydro analogues **100a**–**d** were synthesized in the same manner. Deacetylation of **100b** and **102b** led to the formation of **101** and **103**, respectively. Treatment of two 3-*O*-acyl derivatives with iodomethane in dichloromethane yielded compounds **104a**–**b**, and this was followed by sulfation with pyridine-SO_3_. Thus, betaines **105a**–**b** were synthesized (Scheme 28).

The affinity of M6S to the *μ* (ki = 0.9 ± 0.01 nM), *δ* (ki = 18 ± 0.4 nM), and *κ*-3 (ki = 6.3 ± 0.3 nM) receptors improved slightly compared to morphine (ki *= μ* (2.5 ± 0.3 nM), *δ* (58 ± 3.8 nM), and *κ*-3 (13.9 ± 4.1 nM)). Furthermore, it showed low affinity to *κ*-1 receptors and no affinity for *κ*-2 receptors. Derivative **102a** and its 7,8-dihydro analogue **100a** demonstrated similar activity. As compared to M6S, codeine 6-*O*-sulfate **102i** and its dihydro derivative **100d** showed a significant decline in all opioid receptor affinity. A low affinity of betaines **105a** and **105b** for all receptors was also observed. The 3-*O*-propionyl **102c**, 3-*O*-isobutyryl **102d**, and 3-*O*-pivaloyl **102e** analogues of M6S demonstrated high affinity for *μ*- receptors but low affinity for *κ*-1 receptors while **102c**, as the best analogue, showed four times higher affinity than morphine for *μ*-receptors (K_i_ = 0.6 ± 0.15 nM). It should be noted that, nowadays, the κ-subtype, especially κ-3, no longer has approval [96].

**Scheme 29:**
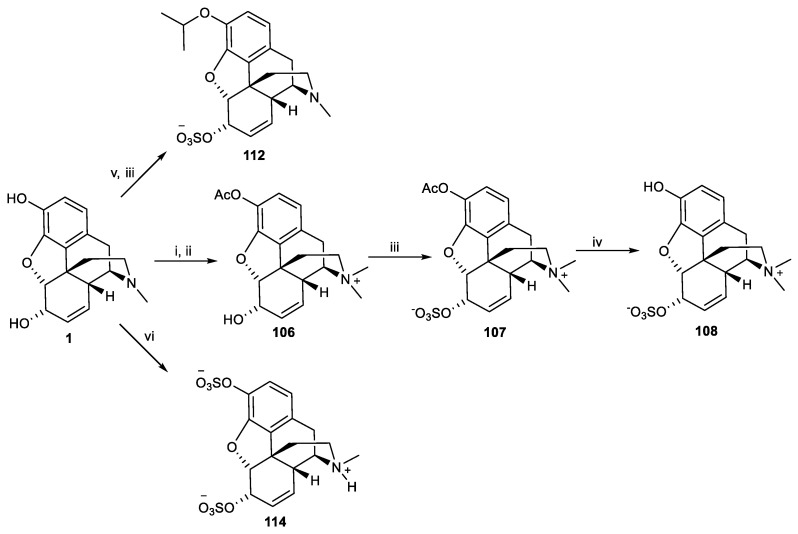
Reagents and conditions: (i) acetic anhydride, NaHCO_3_, H_2_O, r.t, 1 h; (ii) MeI, acetone, 40 °C, 4 h; (iii) pyridine-SO_3_, pyridine, 60 °C, 3.5 h; (iv) 20% aq. K_2_CO_3_ solution, r.t, 1 h; (v) 2-iodopropane, NaOEt, EtOH, reflux, 4 h; (vi) *N*,*N*′-dicyclohexylcarbodiimide, *conc*. sulfuric acid, DMF, 0 °C, 15 min.

**Scheme 30:**
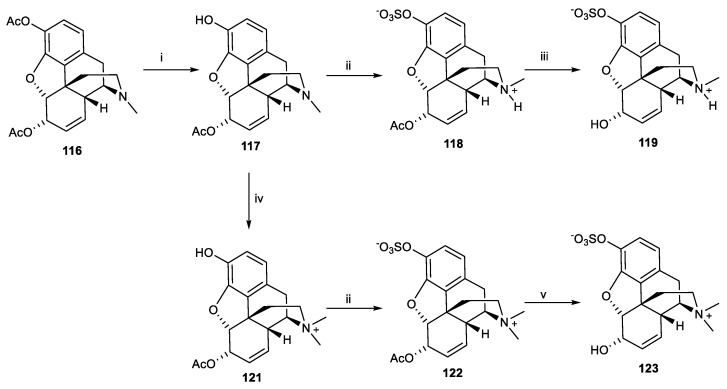
Reagents and conditions: (i) hydroxylamine hydrochloride, EtOH, 60 °C, 45 min; (ii) pyridine-SO_3_, pyridine, 60 °C, 3.5 h; (iii) 10% aq. NaOH, MeOH, r.t, 1 h; (iv) MeI, acetone, 40 °C, 4 h; (v) 20% aq. K_2_CO_3_ solution, r.t, 1 h.

In 2011, Váradi et al. synthesized several 3-*O*-sulfate and 6-*O*-sulfate ester derivatives of morphine, dihydromorphine, codeine, and dihydrocodeine [97]. The same procedure, as already explained, was used for the monosubstitution of ester analogues. Morphine-3,6-*O*-disulfate **114** was prepared directly from the reaction of morphine with *N*,*N*′-dicyclohexylcarbodiimide and concentrated sulfuric acid (Scheme 29). Compounds **112** and **113** were synthesized by alkylation (isopropyl and ethyl) at 3-hydroxyl, and then sulfated by pyridine-SO_3_. For C3-sulfation, first, the C3-acetyl of diacetylmorphine (**116**) was selectively hydrolyzed, then compound **117** was reacted with pyridine-SO3, followed by hydrolysis of C6-acetyl to yield **119** (Scheme 30). The structures of all derivatives are summarized in Table 13. ^1^H- and ^13^C-NMR, as well as CD/ORD (Circular Dichroism/Optical Rotatory Dispersion Spectroscopy) of all derivatives, were analyzed in detail. These data can be utilized to identify various sulfate esters in biological samples. Most sulfate ester derivatives exhibited selective peripheral analgesic effects.

### 5.2. Modifications on N-17, Together with Different Parts of the Structure

**Scheme 31:**
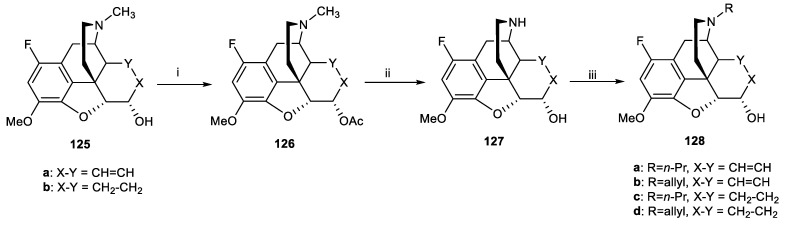
Reagents and conditions: (i) Ac_2_O, reflux, 4 h; (ii) (1) 1-chloroethyl chloroformate, NaHCO_3_, 1,2-dichloroethane, reflux, 8 h; (2) methanol, 50 °C, 1 h; (3) NaOH, ethanol, reflux, 1 h; (iii) RX (*n*-propylbromide or allyl bromide), NaHCO_3_, DMF, 95 °C, 18 h.

In 2015, Hosztafi and Marton [35] synthesized novel *N*-substituted-1-fluoronorcodeine **128a**–**b** and dihydrocodeine derivatives **128c**–**d**. 1-Fluoro-6-*O*-acetylnorcodeine (**126a**) was synthesized from the reaction of 1-fluorocodeine (**125a**) with acetic anhydride to protect the 6-hydroxyl group, and then the demethylation of compound **127a** was performed with 1-chloroethyl chloroformate. Alkyl groups (propyl and allyl) were substituted in *N*-17 moiety to obtain **128a**–**b** (Scheme 31). The corresponding dihydro analogues **128c**–**d** were synthesized in a same way. These derivatives could be potential candidates in pharmacology and can be employed for fluorine-18 labeled morphinan radiotracers as standard references in imaging.

**Scheme 32:**
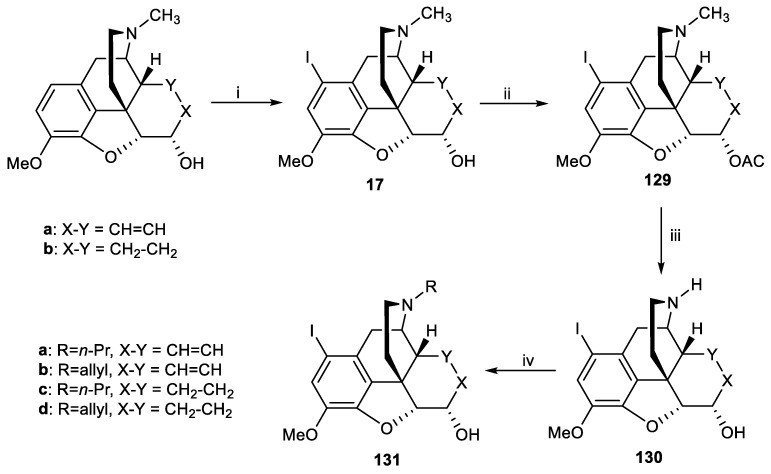
Reagents and conditions: (i) NaI, chloramine T, 0.1 M hydrochloric acid; (ii): Ac_2_O, reflux, 4 h; (iii): (1) α-chloroethyl chloroformate, NaHCO_3_, 1,2-dichloroethane; (2) methanol, 50 °C, 1 h; (3) 5% NaOH, ethanol, reflux, 1 h; (iv): RX (*n*-propylbromide or allyl bromide), NaHCO_3_, DMF, 95 °C, 18 h.

Novel *N*-propyl- and -allyl-substituted derivatives of 1-iodocodeine and their 7,8-dihydro analogues were synthesized in 2018 by Hosztafi et al. [98]. The synthesis of compound **17** was described in the previous section. The reaction of **17** with α-chloroethyl chloroformate followed by realkylation of **130** yielded the corresponding *N*-alkyl analogues **131a**–**d** (Scheme 32). The newly developed derivatives can be applied as a tool for preparing ^18^F-labelled aryl fluorides and can be used as intermediates for the synthesis of palladium aryl complexes, arylstannanes, and aryl-pinacol boronic esters.

**Figure 9:**
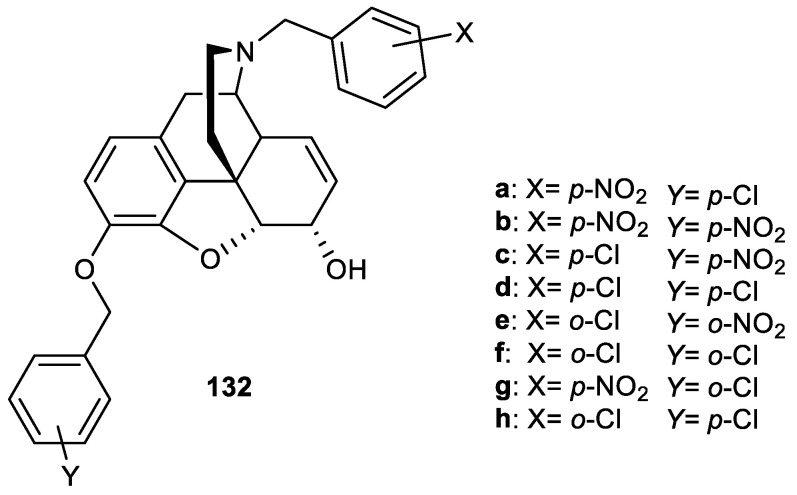
*N*-AIkyl-3-*O*-alkyl derivatives of normorphine.

Shel’metsi et al. synthesized a new series of *N*-benzyl-3-*O*-benzyl- and 3-*O*-benzoylnormorphine analogues and also developed a novel selective method for the 3-*O*-alkylation of morphine, which resulted in the production of various *N*-aIkyl-3-*O*-alkyl-normorphine derivatives [99]. The first step for the preparation of these analogues involved the reaction of normorphine with a variety of substituted-benzyl halides, followed by the Claisen method, where 3-*O*-benzyl-*N*-substituted normorphine derivatives were selectively obtained by reacting the phenolic hydroxide group with alkyl halides in the presence of potassium carbonate in absolute acetone (Figure 9). Among these compounds, 3-(*p*-nitrobenzyl)-*N*-(*p*-nitrobenzyl)normorphine (**132b**) demonstrated the most potent analgesic effect without undesirable psychomimetic side effects.

**Scheme 33:**
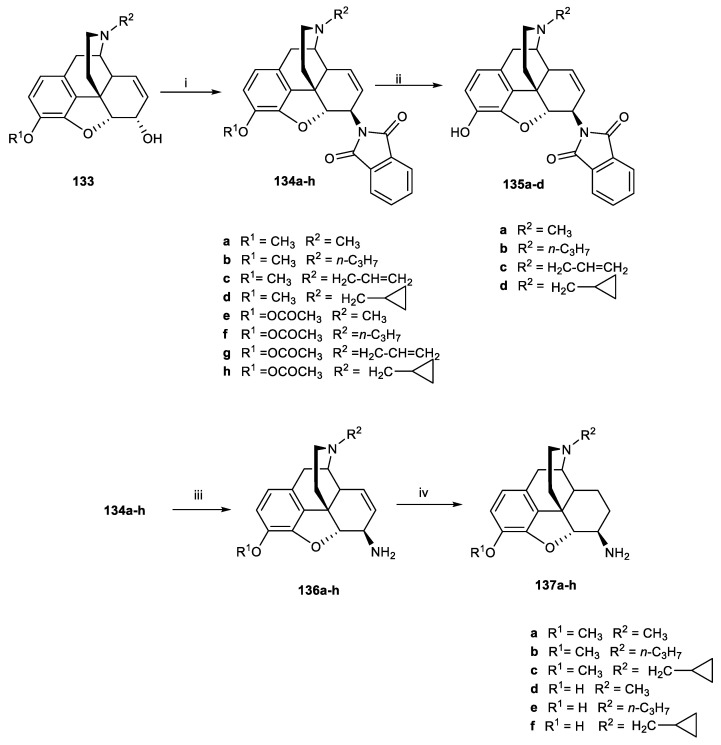
Reagents and conditions: (i) PPh_3_, phthalimide, diethyl azodicarboxylate, benzene; (ii) hydroxylamine hydrochloride, EtOH, 50 °C, 10 min; (iii) 98% hydrazine hydrate, EtOH; (iv) Pd/C 10%, EtOH.

**Scheme 34:**
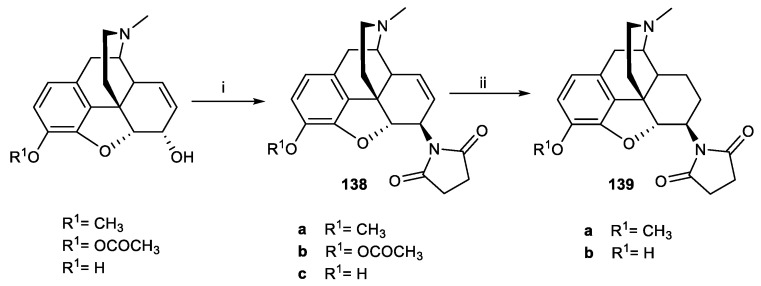
Reagents and conditions: (i) PPh_3_, succinimide, diethyl azodicarboxylate, benzene; (ii) Pd/C 10%, EtOH.

By the use of the Mitsunobu reaction for the alkylation of cyclic imides (phthalimido and succinimido) with various alcohols, Simon et al. synthesized new 6*β*-aminomorphine and codeine analogues [100]. Through the reaction of 3-*O*-acetyl-*N*-substituted derivatives of morphine and codeine with phthalimide or succinimide utilizing triphenylphosphine and diethyl azodicarboxylate, 6*β*-phthalimido **134a**–**h** or succinimido **138a**–**c** derivatives were synthesized. Then, phthalimido-substituent derivatives **134a**–**h** were converted into the corresponding primary amine compounds **136a**–**h** by treatment with hydrazine hydrate in ethanol (Scheme 33). The Δ^7,8^ double bond in ring C of succinimido derivatives was hydrogenated with Pd/C to yield **139a**–**b** (Scheme 34). Such a reaction was not possible with the corresponding phthalimido analogues.

**Scheme 35:**
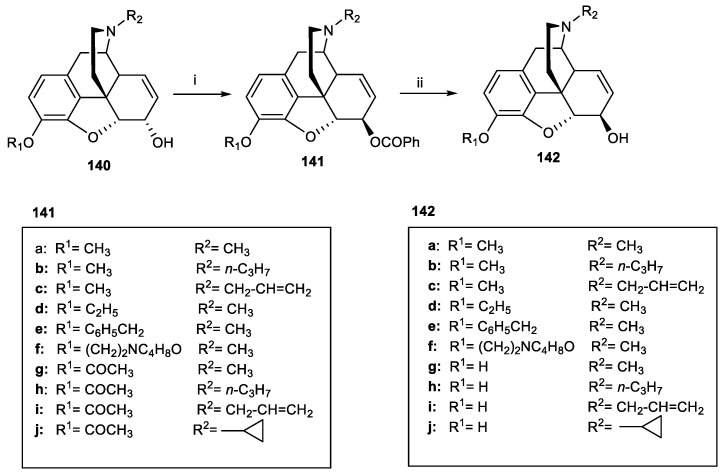
Reagents and conditions: (i) PPh_3_, benzoic acid, anhydrous benzene, diethyl azodicarboxylate, 1 h; (ii) 10% aqueous KOH, EtOH, reflux, 10 min.

Isocodeine **142a** and isomorphine **142g** are the epimers of codeine and morphine, respectively, which have opposite stereochemistry at the C6 hydroxyl group. Several approaches have been reported to synthesize **142a** and **142g**. However, only few reports have been published on their novel derivatives. In 1991, Simon et al. reported novel analogues of isocodeine and isomorphine by the employment of the Mitsunobu reaction [101]. The reaction was started from different *N*-substituted analogues of isocodeine and isomorphine. In the first step, **140** reacted with triphenylphosphine and diethyl azodicarboxylate to obtain **141a**–**j**. In the second step, the ester was hydrolyzed to hydroxy at the 6-position with good to excellent yield to obtain the final derivatives **142a**–**j** (Scheme 35).

**Scheme 36:**
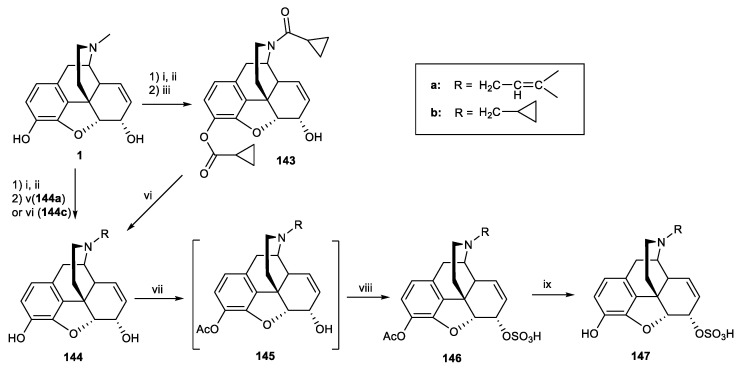
Reagents and conditions: (i) acetic anhydride; (ii) BrCN, HCl; (iii) cyclopropanecarbonyl chloride, CHCl_3_, triethylamine, reflux, 8 h; (iv) LiAlH_4_, THF, r, t; (v) dimethylallyl bromide, K_2_CO_3_, DMF, 90–95 °C, N_2_, 3 h; (vi) allyl bromide, Na_2_CO_3_, dry DMF, 16 h, 90 °C; (vii) Ac_2_O, aq.NaHCO_3_, 30 min; (viii) ClSO_3_H, dry pyridine; (ix) 5% NaOH-MeOH.

It has already been proven that glucuronidation and sulfation of morphine at the 6-position dramatically improved the analgesic activity. On the other hand, it is noteworthy to explain what occurs when an allyl or a cyclopropylmethyl group is attached to nitrogen. Neither naltrexone nor naloxone has any analgesic effect, and nalorphine has only mild analgesic activity. In this regard, Hirano et al. synthesized two novel *N*-substituted normorphine derivatives **147a**–**b** that conjugated to the sulfate group at the 6-position of the morphine scaffold (Scheme 36) and evaluated their analgesic and antagonistic activities [102]. Evaluation of analgesic activity demonstrated that *N*-cyclopropylmethylnormorphine-6-sulfate **147b** and *N*-dimethylallylnormorphine-6-sulfate **147a** had similar analgesic properties to *N*-cyclopropylmethylnormorphine **144b** and *N*-dimethylallylnormorphine **144a** due to the acetic acid writhing test in mice. Compounds **147a** and **147b** showed higher antagonistic activity than their parent compounds to morphine analgesia by tail pinch test in mice.

Simon et al. synthesized several *N*-substituted-C1-halogenated derivatives of morphine **148a**–**d**, **148e**–**h** codeine, and their dihydro analogues **148i**–**l** [29]. The synthesis of C1-halogenation derivatives was explained in the previous sections. *N*-substituted (allyl and *n*-propyl) analogues were synthesized according to the von Braun–Olofson method (Table 14) [103].

**Scheme 37:**
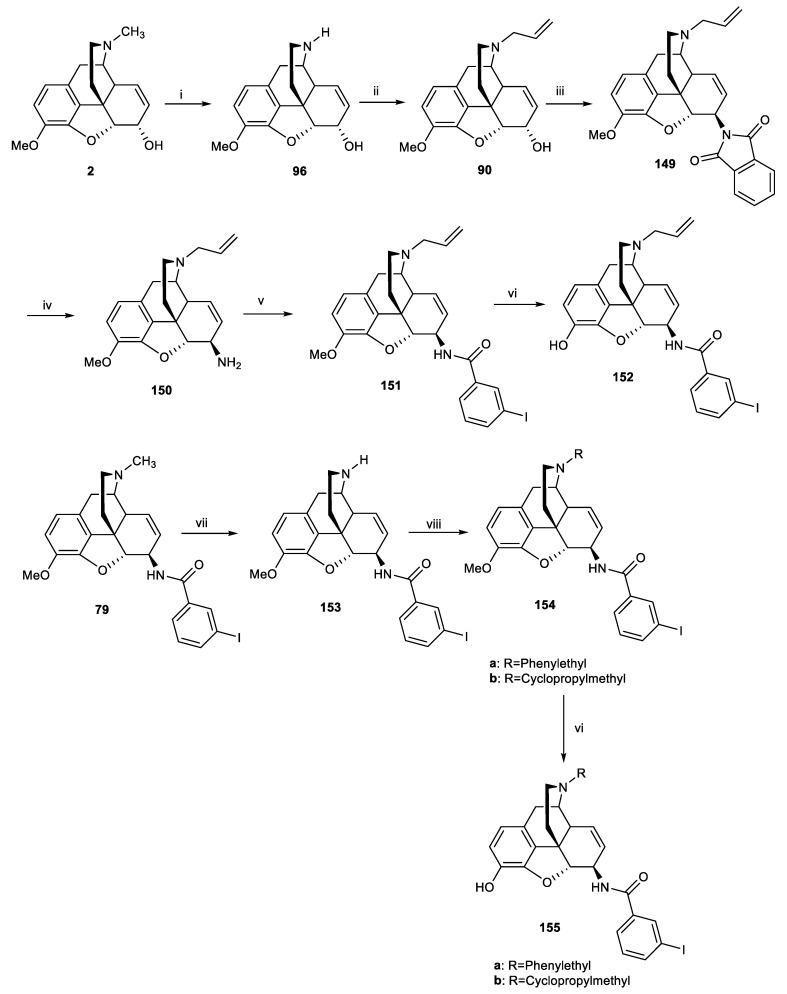
Reagents and conditions: (i) chloroethyl chloroformate, NaHCO_3_, 1,2-dichloroethane, 30 min, r.t, then 16 h, 85 °C; (ii) allyl bromide, Na_2_CO_3_, dry DMF, 16 h, 90 °C, 68%; (iii) phthalimide, Ph_3_P, dry THF, DIAD in dry toluene, 16 h, r.t, 68%; (iv) hydrazine hydrate, *cis*-2-penten-1-ol, MeOH, 16 h, r.t, 100%; (v) 3-iodobenzoic acid, HATU, *N*,*N*-diisopropylethylamine (DIEA), dimethyl formamide (DMF), 15 min, r.t, 70%; (vi) BBr_3_, DCM, 30 min, 0 °C to r.t, 50–60%; (vii) DIAD, acetonitrile, 20 h, 65 °C, then pyridine-HCl, 72 h, r.t, 54%; (viii) alkyl halide, Na_2_CO_3_, or Cs_2_CO_3_, DMF, 16 h, 90 °C.

According to our discussion in the previous sections to modify the scaffold of IBNtxA (**78**) (Figure 4), Váradi et al. synthesized potent analgesic analogues of 3-iodobenzoyl naltrexamine (IBNtxA) by substituting a new alkyl and allyl group in nitrogen position 17 instead of a cyclopropylmethyl, along with the incorporation of a 7,8-double bond and removal of the 14-hydroxy group (Scheme 37) [75]. The affinity of compounds **155a** and **155b** for the 6TM/E11 site decreased compared with **78**. However, both compounds showed an increased affinity for MOR-1, DOR-1, and KOR-1. The affinity of compound **152** for MOR-1, KOR-1, and DOR-1 receptors was higher than 6TM/E11. In addition, the affinity of **152** for these receptors compared to the similar derivative with the hydroxy group at position 14 was increased (Table 15). Compound **155b** demonstrated 15-fold greater potency than morphine in tail flick analgesic tests (ED_50_ = 0.33 ± 0.09 mg/kg). Compound **155b** was selected for [35S]GTPγS binding assay and was found to be a full agonist for MOR and a dual agonist at KOR-1 and DOR-1 receptors (EC_50_ = 0.027−0.41 nM for mentioned receptors). Biological studies also showed that pretreatment with compound **155b** prevented cocaine-associated reward behaviors. In conclusion, *N*-17 alkylation, removal of the 14-OH, and incorporation of a 7, 8 double bond lead to dual kappa–delta agonism, which could be an ideal starting point for novel analgesics and treatments for cocaine addiction.

## 6. Conclusions

There have been extensive studies on the chemistry of morphine and codeine and their congeners for several decades. Efforts to modify their structures to obtain analogues with fewer undesirable side effects continue. These changes have led to the synthesis of potent analogues such as oxycodone, naltrexone, and buprenorphine, which are used as drugs. However, the semi-synthesis starting from morphine and codeine main scaffolds is still of interest. Some newly synthesized analogues have shown better biological effects than morphine with fewer side effects and, in some cases, changed morphine’s agonist activity to antagonist. There are many reviews about morphine and codeine metabolism in the body and related pharmaceutical studies. This review spotlights the majority of morphine and codeine derivatives synthesized to date and summarizes the most effective analogues in biological investigations. Several studies have demonstrated that the C3-hydroxyl group plays a significant role in interacting with opioid receptors, and substituting different groups at this position did not work in most cases. Even the substitution of amine with C3-hydroxyl decreased its affinity for *μ*, *δ*, and *κ* receptors. This explains why codeine derivatives do not provide better effects than morphine derivatives. However, structural changes in C6 and *N*-17 positions have helped to improve the properties of synthetic derivatives compared to codeine. The C6-hydroxyl and *N*-17 are considered significant parts of morphine and codeine’s structures. Modifications of these moieties can lead to the synthesis of much more potent analogues with decreased side effects. A review of the synthetic derivatives reported here indicated that replacing C6 hydroxyl with some groups, especially acrylamide groups, can enhance opioid receptor affinity as well as analgesic activity. The change in this position, along with the substitution of different groups in the *N*-17 position, have the ability to increase the analgesic effect. By further studying these structural changes, it can be of hope that in the future, novel derivatives of morphine will show higher analgesic activity and be devoid of some dangerous side effects of morphine. Hopefully, this review will lead to further synthesis of more potent analogues or more studies of the pharmacological properties of some reported derivatives.

## Figures and Tables

**Table 1:** Potencies of morphine and analogues at *μ*, *δ*, and *κ*-Opioid receptor recognition sites labeled by [^3^H]DAMGO, [^3^H]DPDPE, and [^3^H]U69593, respectively, in the guinea pig brain minus cerebellum (*μ* and *δ*) and the guinea pig cerebellum (*κ*).

IC_50_ (nM)			
Compound	[^3^H]DAMGO (*µ*)	[^3^H]DPDPE (*δ*)	[^3^H]U69593 (*κ*)
**Morphine**	8.5	430	130
**40a**	2600	31% at 10 μM	9500
**40b**	200	7400	910
**40c**	43% at 10 μM	16% at 10 μM	76,000
**40d**	49% at 10 μM	17% at 10 μM	25,000
**40e**	3300	10% at 10 μM	15,000
**40f**	41% at 10 μM	14% at 10 μM	126,000
**41**	640	7900	5100

**Table 2:** Opioid receptor binding data for compounds **46a**–**e**.

	K_i_ (nM ± SE)			Receptor Selectivity ^a^		
Compound	[^3^H]DAMGO (*µ*)	[^3^H]naltrindole (*δ*)	[^3^H]U69593 (*κ*)	*µ:δ*	*µ:κ*	*κ:δ*
**Morphine**	0.88 ± 0.14	140 ±18	24 ± 2.3	156	27	6
**46a**	53 ± 3.0	2400 ± 190	740 ± 75	45	14	3
**46b**	63 ± 15	5700 ± 1100	2800 ± 420	90	44	2
**46c**	240 ± 16	1600 ± 110	290 ± 8.1	7	1	6
**46d**	59 ± 3.7	1500 ± 100	240 ± 23	25	4	61
**46e**	33 ± 5.1	5500 ± 190	3400 ± 540	67	103	2

^a^ Receptor selectivity is expressed as the ratio of the corresponding K_i_ values.

**Table 3:** K_i_ value inhibition of *δ*, *κ*, and *µ* opioid binding to Chinese hamster ovary membranes by compound **50** ^a^.

	K_i_ ± SEM (nM)			Selectivity	
Compound	[^3^H]DAMGO (*µ*)	[^3^H]naltrindole (*δ*)	[^3^H]U69593 (*κ*)	*µ:κ*	*δ:κ*
**Morphine**	0.88 ± 0.14	140 ±18	24 ± 2	0.04	5.8
**Levorphanol**	0.21 ± 0.02	4.2 ± 2.3	2.3 ± 0.3	0.09	2
**50**	30 ± 3	32 ± 3	220 ± 10	0.13	0.14

^a^ Chinese hamster ovary membrane, 0.5 mg of protein/sample, were incubated with 12 different concentrations of the compound in the presence of receptor-specific radioligands at 25 °C, in a final volume of 1 mL of 50 mM Tris-HCl, pH 7.5.

**Table 4:** The relative analgesic activity of azidomorphine and azidocodeine.

Compound	Hot Plate Test ED_50_ (mg/kg)	Tail Flick Test ED_50_ (mg/kg)
**Morphine**	4.7	1.8
	(2.9–7.8) ^a^	(1.08–3.15)
**Codeine**	14	22
	(6.7–29.4)	(15.3–28.8)
**67a**	0.016	0.012
	(0.007–0.038)	(0.009–0.016)
**67b**	0.36	0.61
	(0.16–0.79)	(0.4.1–0.79)

^a^ Figures in parentheses indicate 95% confidence limits.

**Table 5:** K_i_ inhibition values of *μ*, *δ*, and *κ* opioid binding to CHO membranes, and EC_50_ values of stimulation of [^35^S]GTPγS binding by amides **72a**–**h** and **73** mediated by *μ*, *δ*, and *κ* opioid receptors.

	K_i_ ± SEM (nM)			EC_50_		
Compound	[^3^H]DAMGO (*µ*)	[^3^H]DPDPE (*δ*)	[^3^H]U69593 (*κ*)	*µ*	*δ*	*κ*
**Morphine**	1.1 ± 0.1	140 ± 2	46.9 ± 14			
**M6G**	12.85 ± 0.95	160.96 ± 0.73	4058.75 ± 230	72.3 ± 26.7	190.35 ± 22.9	>10K
**72a**	0.35 ± 0.08	9.5 ± 2.6	0.96 ± 0.2	1.7 ± 0.2	18.8 ± 2.2	4.8 ± 0.1
**72b**	0.59 ± 0.19	8.89 ± 2.1	2.84 ± 0.96	4.8 ± 2.1	25.2 ± 3.0	5.0 ± 1.0
**72c**	0.21 ± 0.07	8.96 ± 1.95	2.65 ± 1.25	2.8 ± 0.2	37.8 ± 1.9	9.8 ± 0.02
**72d**	0.40 ± 0.03	24.95 ± 2.33	4.13 ± 1.21	5.5 ± 0.9	62.7 ± 24.0	5.3 ± 1.3
**72e**	0.23 ± 0.05	3.39 ± 0.03	1.53 ± 0.2	2.4 ± 0.3	6.20 ± 0.42	11.22 ± 3.51
**72f**	0.20 ± 0.04	18.0 ± 5.63	2.63 ± 1.1	1.9 ± 0.2	24.5 ± 3.0	1.2 ± 0.1
**72g**	0.20 ± 0.04	0.94 ± 0.02	0.75 ± 0.2	0.1 ± 0.0	1.3 ± 0.3	0.03 ± 0.02
**72h**	0.33 ± 0.02	14.42 ± 1.26	0.58 ± 0.17	6.0 ± 2.1	32.4 ± 2.1	4.5 ± 0.2
**73**	19.92 ± 4.29	50.78 ± 8.86	>10K	65.1 ± 21.8	74.0 ± 12.7	5096

**Table 6:** Receptor binding and in vivo analgesia data of selected 6*β*-acylaminomorphinans (**75a**–**d**) ^a^.

	Affinity (K_i_ (nM) ± SEM)			Analgesia (ED_50_, mg/kg, s.c.)
Compound	MOR-1	KOR-1	DOR-1	
**Morphine**	4.60 ± 1.81			4.96 ± 0.96
**75a**	0.10 ± 0.02	2.90 ± 0.66	10.26 ± 6.76	3.13 ± 1.09
**75b**	0.15 ± 0.03	1.97 ± 0.01	9.38 ± 1.53	>10
**75c**	0.19 ± 0.09	10.52 ± 0.90	14.52 ± 6.82	>10
**75d**	0.74 ± 0.12	5.43 ± 1.38	15.26 ± 1.74	>10
**75e**	0.12 ± 0.006	0.81 ± 0.09	5.15 ± 0.75	>10

^a^ Competition studies were performed with the indicated compounds against ^125^I-BNtxA (0.1 nM) in membranes from CHO cells stably expressing the indicated cloned mouse opioid receptors.

**Table 7:** Binding and analgesia of **79** and **80**.

	K_i_ (nM) ± SEM ^a^				Tail Flick Analgesia (CI) ^b^, ED_50_ [mg/kg]
Compound	MOR-1	KOR-1	DOR-1	6TM/E11	
**Morphine**	4.6 ± 1.81			>1000	4.96 ± 0.96
**IBNtxA**	0.11 ± 0.02	0.03 ± 0.001	0.24 ± 0.05	0.16 ± 0.04	0.39 (0.15, 0.58)
**79**	1.2 ± 0.48	10 ± 3.5	49 ± 6.4	61 ± 7.7	>10
**80**	0.037 ± 0.0051	0.21 ± 0.046	0.88 ± 0.08	27 ± 18	6.31 (4.6, 8.5)

^a^ [^125^I]BNtxA (0.1 nM) competition binding assays were performed in membranes prepared from CHO cells expressing mouse MOR-1, DOR-1, or KOR-1, or mouse brain in the presence of CTAP, DPDPE, and U50,488 blockers at 200 nM. ^b^ Assays were performed at least two times, and means ± SEM of replicates or CIs were reported.

**Table 8:** In vitro efficacy of the compounds **82a**–**d** in the [^35^S]GTPγS assay ^a^.

MOR			KOR	
Compound	EC_50_ (nM)	%E_max_	EC_50_ (nM)	%E_max_
82a	4.62 ± 0.28	114.36 ± 7.06	nd *^b^*	nd *^b^*
82b	2.83 ± 0.41	113.63 ± 4.54	nd *^b^*	nd *^b^*
82c	8.16 ± 2.64	114.85 ± 1.26	nd *^b^*	nd *^b^*
82d	7.98 ± 2.35	117.16 ± 1.98	58.83 ± 7.49	87.88 ± 4.9
DAMGO	19 ± 7.0	nd *^b^*	nd *^b^*	nd *^b^*
U50,488H	nd *^b^*	nd *^b^*	17 ± 6.1	nd *^b^*

^a^ Functional properties were studied using agonist-induced stimulation of the [^35^S]GTPγS binding assay. Potency is represented as EC_50_ (nM) and efficacy as percent maximal stimulation (%E_max_) relative to standard agonist DAMGO (MOR), or U50,488H (KOR)at 100 nM. *^b^* Not determined.

**Table 9:** *N*-substituted normorphine derivatives.

Compound		R^1^	R^2^	R^3^
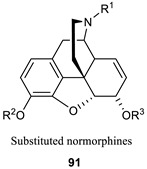	**a**	CH_2_CH_2_CH_3_	H	H
	**b**	CH_2_CONH_2_	H	H
	**c**	CH_2_COOEt	H	H
	**d**	CH_2_CH_2_OC_6_H_5_	H	H
	**e**	CH_2_CH=CH_2_CH_2_Br	H	H
	**f**	CH_2_CHBr=CH_2_	H	H
	**g**	CH_2_CH_2_CH_3_	CH_3_	H
	**h**	CH_2_(CH_3_)_2_	H	H
	**i**	CH_2_(CH_3_)_2_	CH_3_	H
	**j**	CH_2_CH_2_CN	H	H
	**k**	CH_2_CHOHCH_3_	H	H
	**l**	CH_2_CH_2_COOEt	H	H
	**m**	CH_2_C(CH_3_)=CH_2_	H	H
	**n**	CH_2_CH(CH_3_)_2_	CH_3_	H
	**o**	CH_2_(CH_2_)_3_CH_3_	H	H
	**p**	CH_2_C_6_H_5_	H	H
	**q**	CH_2_COC_6_H_5_	H	H
	**r**	CH_2_CH_2_C_6_H_5_	H	H

**Table 10:** *N*-substituted dihydronormorphine derivatives.

Compound		R^1^	R^2^	R^3^
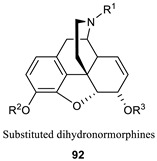	**a**	CH_2_CH=CH_2_	CH_3_	H
	**b**	CH_2_CH=CH_2_	Ac	Ac
	**c**	CH_2_CH_2_CH_3_	CH_3_	H
	**d**	CH_2_CH_2_CH_3_	Ac	Ac
	**e**	CH_2_C(CH_3_)=CH_2_	H	H
	**f**	CH_2_CH(CH_3_)_2_	H	H

**Table 11:** Opioid receptor binding affinity and selectivity of compounds **93a** and **94** at MOP, DOP, and KOP receptors.

	K_i_ ± SEM (nM)			Selectivity	
Compound	MOP	DOP	KOP	*δ:μ*	*κ:μ*
**Morphine**	6.556 ± 0.74	2176 ± 19	1136 ± 9	33	17
**93a**	0.936 ± 0.14	37.06 ± 5.5	1076 ± 18	40	115
**94**	79.56 ± 1.1	8696 ± 171	5656 ± 24	11	7

Binding assays were performed with membranes from rat brain (MOP and DOP receptors) and guinea pig brain (KOP receptors).

**Table 12:** Values of K_i_ and IC_50_ of various ligands in radioligand binding assay and ED_50_ by in vivo studies of synthetic compounds.

	Affinity		Analgesia
Compound	IC_50_^a^ (%95 CI ^b^) (nM)	K_i_^c^ (nM)	ED_50_^d^ (95% CI) (mg/kg)
**Codeine**	327.4	144.2	9.55
	(152.5 to 702.9)	(67.1 to 309.6)	(8.50 to 10.74)
**98d**	363.1	159.9	8.68
	(150.6 to 875.0)	(66.3 to 385.4)	(6.47 to 11.66)
**98c**	442.7	195.0	17.05
	(255.3 to 767.7)	(112.4 to 338.1)	(13.72 to 22.49)
**98a**	486.6	214.3	17.57
	(381.3 to 620.9)	(167.9 to 273.5)	(14.56 to 19.97)
**98f**	559.3	246.3	26.14
	(299.0 to 1046.0)	(131.7 to 460.7)	(19.82 to 34.46)
**98r**	536.7	236.4	30.27
	(349.3 to 824.6)	(153.8 to 363.2)	(19.41 to 47.21)
**98h**	566.3	249.4	31.17
	(274.7 to 1167.0)	(121.0 to 514.0)	(22.98 to 42.28)

^a^ IC_50_, the half maximal inhibitory concentration, ^b^ CI, confidence interval, ^c^ K_i_, affinity of the ligand, ^d^ ED_50_, the dose (or concentration) causing 50 % of maximum effect.

**Table 13:** Structures of synthesized compounds.

Compound		R^1^	R^2^	R^3^	C7–C8 Bond
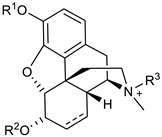	**108**	H	SO_3_^−^	CH_3_	Double
	**109**	H	SO_3_^−^	CH_3_	Single
	**110**	CH_3_	SO_3_^−^	CH_3_	Double
	**111**	CH_3_	SO_3_^−^	CH_3_	Single
	**112**	^i^Pr	SO_3_^−^	H	Double
	**113**	Et	SO_3_^−^	H	Double
	**114**	SO_3_^−^	SO_3_^−^	H	Double
	**115**	CH_3_	SO_3_^−^ (6β)	H	Double
	**119**	SO_3_^−^	H	H	Double
	**120**	SO_3_^−^	H	H	Single
	**123**	SO_3_^−^	H	CH_3_	Double
	**124**	SO_3_^−^	H	CH_3_	Single

**Table 14:** Structures of **148a**–**l** derivatives.

Compound		X	R^1^	R^2^	C7–C8 Bond
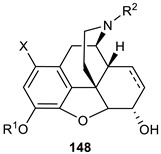	**a**	Br	H	CH_2_CH=CH_2_	Double
	**b**	Cl	H	CH_2_CH=CH_2_	Double
	**c**	Br	H	*n*-Pr	Double
	**d**	Cl	H	*n*-Pr	Double
	**e**	Br	CH_3_	CH_2_CH=CH_2_	Double
	**f**	Cl	CH_3_	CH_2_CH=CH_2_	Double
	**g**	Br	CH_3_	*n*-Pr	Double
	**h**	Cl	CH_3_	*n*-Pr	Double
	**i**	Br	H	CH_2_CH=CH_2_	Single
	**g**	Cl	H	CH_2_CH=CH_2_	Single
	**k**	Br	CH_3_	CH_2_CH=CH_2_	Single
	**l**	Cl	CH_3_	CH_2_CH=CH_2_	Single

**Table 15:** Binding and Analgesia of compounds **152**, **155a**–**b**.

	K_i_^a^ (nM) ± SEM				Tail Flick Analgesia (CI) ^b^, ED_50_ [mg/kg]
Compound	MOR-1	KOR-1	DOR-1	6TM/E11	
**Morphine**	4.6 ± 1.81			>1000	4.96 ± 0.96
**152**	0.034 ± 0.006	0.022 ± 0.0043	0.39 ± 0.0041	0.78 ± 0.1	0.53 (0.45, 0.59)
**155a**	0.088 ± 0.028	0.14 ± 0.022	0.13 ± 0.032	14 ± 3.2	1.3 (1.03, 2.2)
**155b**	0.021 ± 0.0034	0.0064 ± 0.002	0.08 ± 0.019	0.47 ± 0.009	0.33 ± 0.09
**IBNtxA**	0.11 ± 0.02	0.03 ± 0.001	0.24 ± 0.05	0.16 ± 0.04	0.39 (0.15, 0.58)

^a^ [^125^I]BNtxA (0.1 nM) competition binding assays were performed in membranes prepared from CHO cells expressing mouse MOR-1, DOR-1, or KOR-1, or mouse brain in the presence of CTAP, DPDPE, and U50,488 blockers at 200 nM. ^b^ Assays were performed at least two times, and means ± SEM of replicates or CIs were reported.

## Data Availability

Not applicable.
